# Functional Bread Produced in a Circular Economy Perspective: The Use of Brewers’ Spent Grain

**DOI:** 10.3390/foods12040834

**Published:** 2023-02-15

**Authors:** Antonietta Baiano, Barbara la Gatta, Mariacinzia Rutigliano, Anna Fiore

**Affiliations:** Dipartimento di Scienze Agrarie, Alimenti, Risorse Naturali e Ingegneria (DAFNE), University of Foggia, 71122 Foggia, Italy

**Keywords:** brewers’ spent grain, bread-making, circular economy, common wheat, dietary fibre, durum wheat, emmer, phenolics, proteins, sustainable food production

## Abstract

Brewers’ spent grain (BSG) is the main by-product of the brewing industry, corresponding to ~85% of its solid residues. The attention of food technologists towards BSG is due to its content in nutraceutical compounds and its suitability to be dried, ground, and used for bakery products. This work was aimed to investigate the use of BSG as a functional ingredient in bread-making. BSGs were characterised for formulation (three mixtures of malted barley and unmalted durum (Da), soft (Ri), or emmer (Em) wheats) and origin (two cereal cultivation places). The breads enriched with two different percentages of each BSG flour and gluten were analysed to evaluate the effects of replacements on their overall quality and functional characteristics. Principal Component Analysis homogeneously grouped BSGs by type and origin and breads into three sets: the control bread, with high values of crumb development, a specific volume, a minimum and maximum height, and cohesiveness; Em breads, with high values of IDF, TPC, crispiness, porosity, fibrousness, and wheat smell; and the group of Ri and Da breads, which have high values of overall smell intensity, toasty smell, pore size, crust thickness, overall quality, a darker crumb colour, and intermediate TPC. Based on these results, Em breads had the highest concentrations of nutraceuticals but the lowest overall quality. Ri and Da breads were the best choice (intermediate phenolic and fibre contents and overall quality comparable to that of control bread). Practical applications: the transformation of breweries into biorefineries capable of turning BSG into high-value, low-perishable ingredients; the extensive use of BSGs to increase the production of food commodities; and the study of food formulations marketable with health claims.

## 1. Introduction

Topics related to a circular and sustainable economy are gaining more and more attention as means to combat climate change, reduce the use of fossil fuels and other natural resources, recover all products from resources without generating waste, and thus creating new opportunities for long-term economic growth [1]. In this perspective, the sustainable management of by-products and waste is an integral aspect of the circular economy.

Brewers’ spent grain (BSG) is a lignocellulosic material representing the main by-product generated from the brewing industry, since it corresponds to about 85% of the total solid residues [2]. It has been estimated that between 14 and 20 kg of wet BSG are obtained per 100 L of finished beer [3]. The average annual global production is estimated to be around 39 million tons, with ~3.4 million tons (which could become 8.5 be the end of 2030) produced in the European Union [4]. In Italy, a production of 188 thousand tons/year of BSG is estimated. Thirty percent of them are reused, mainly in the livestock sector. Furthermore, the BSG market value as cattle feed is very low, being around ~€35/ton [5]. The remaining 70% must be disposed of by breweries, with costs between 75 and 100 €/t (in the EU) and environmental impacts deriving from the release of 513 kg of CO_2_ equivalent of greenhouse gases per ton of landfilled BSG [6]. The research of Bolwig et al. [7] highlighted that an increasing number of farmers are refusing to use BSG as animal feed. This situation, together with the absence of on-site storage facilities, slows down the BSG disposal, sometimes forcing breweries to halt production.

The composition of BSG paves the way for the possibility of recovering and recycling them as secondary starting materials to produce a variety of value-added goods, included functional foods. Although BSG composition depends on several factors including the type of grains (mainly malted barley and wheat, but also unmalted grains and other starchy raw materials) and the malting and mashing methods [8], it includes on average: hemicellulose (~20%), cellulose (15.2–28.7%), lignin (3.35–21%), proteins (18.5–24.7%), lipids (8.4%), starch (5.3%), and ash (3.7%) [9]. From a nutritional standpoint, the interest towards BSG is mainly due to its content in phenolic compounds and soluble and insoluble fibre components such as β-glucans, and the less known arabinoxylans. Since most of the phenolic compounds of cereals are contained in the husk, BSG is rich in valuable antioxidant compounds such as phenolic acids (both benzoic and cinnamic acid derivatives), flavonoids, tannins, proanthocyanidins, and amino-phenolics and, for this reason, can be considered a suitable target for exploitation as a health promoting food supplement [10,11]. β-glucans and arabinoxylans are known to exert physiological functions in the gastro-intestinal tract. In particular, β-glucans are implicated in lowering plasma cholesterol and reducing glycaemic index and the risk of colon cancer, while arabinoxylans exert positive effects such as the production of short-chain fatty acids, the reduction of serum cholesterol, the enhancement of calcium and magnesium adsorption and, because of their esterification with hydroxycinnamic acids, they may also have some antioxidant properties [12]. Regardless of their health effects, the incorporation of arabinoxylans or β-glucans into wheat flour can also negatively influence bread-making. Loaf volume is depressed as a consequence of the effects of gluten dilution; the disruption of the gluten network related to the presence of fibres; the ability of β-glucans to bind water thus reducing its availability for the gluten network development; and, consequently, the gas holding capacity [13,14]. Although the use of BSG in bread-making has been extensively investigated, most research has been focused on the effects of the level of addition of spent grains on the characteristics of doughs and breads [15,16,17,18]. Topics related to the effects of nature (composition and brewing style) and geographical origin of BSG on concentrations of functional compounds in both spent grains and final products have been thoroughly investigated in very few studies and generally not together [19,20].

Our work was aimed at investigating the feasibility of using BSGs from the brewing of Belgian style white beers as functional ingredients in bread-making from a perspective of the sustainable use of natural resources, the reduction of waste disposal, and the re-use of by-products. BSGs were firstly characterised in order to evaluate their content in nutraceutical compounds as a function of both formulation (three different mixtures of malted barley and unmalted cereals) and geographical origin (two different places of cereal cultivation) of the starting brewing materials. To our knowledge, this is the first time that such an approach has been addressed. Successively, the bread samples obtained by replacing the Manitoba wheat flour with two different percentages of each BSG flour were submitted to physical, chemical, image, and sensory analyses with the aim of evaluating the effects of such replacements on their overall quality and functional characteristics.

## 2. Materials and Methods

### 2.1. Production of Cereals Used in Brewing

Barley malt cv. Fortuna was supplied by Agroalimentare Sud (Melfi, Potenza, Italy). The unmalted cereals, i.e., durum wheat (*Triticum durum*) cv. Dauno III, soft wheat (*Triticum aestivum*) cv. Risciola, and emmer (*Triticum dicoccum*) were produced from seeds supplied by CREA-CI Research Centre for Cereal and Industrial Crops (Foggia, Italy) cultivated under the same agricultural practices in two areas of Puglia region, namely Daunia (Soc. Cooperativa Agricola Valleverde, Bovino, Foggia, Italy) and Salento (Birra Salento Societa’ Semplice Agricola Di Leo Consolata and Co, Leverano, Lecce, Italy). The two areas strongly differ for geographical position and climate as follows:

Bovino: Height above sea level: 620 m; Latitude 41.250944 N; Longitude 15.342204 E; Climate zone: E; Degree days: 2243;
Leverano: Height above sea level: 37 m; Latitude 40.289737 N; Longitude 18.001176 E; Climate zone: C; Degree days: 1197.

### 2.2. Brewing Procedures and Obtainment of BSG Flour

BSGs were collected after mashing during brewing of craft Belgian style white beers performed in a 30 L Braumeister system (Speidel Tank-und Behälterbau GmbH, Ofterdingen, Germany) as follows: cereal mixtures made of 65% barley malt and 35% unmalted cereals were crushed with a 2-roller mill (Albrigi Luigi, Stallavena, Verona, Italy) under mill gaps of 0.5 ± 0.1 mm; water-to-cereal ratio was set to 20:5 (*v*/*w*); mash-in temperature was 52 °C, followed by a 13 min stand at 55 °C, a 35 min stand at 64 °C, a 20 min stand at 72 °C, and a final mash-off at 78 °C for 15 min; 7 L of sparge water at 80 °C was passed through the grain bed. Table 1 lists the resulting six types of BSGs differing for type and geographical origin of the unmalted cereals in the mixtures.

Since freezing techniques are not appropriate to preserve BSG quality as they affect its arabinose content [21], after separation of wort, BSG samples were dried at 20 °C for 24 h through a Forced-air-drying system. The dried BSGs were ground (particle size distribution: <15% higher than 500 μm; 35–45% between 500 and 250 μm; 30–40% between 250 and 125 μm; <15% between 125 and 63 μm; <1% lower than 63 μm) with a blade mill, taking care to keep the temperature below 35 °C. The resulting BSG flours were sealed in polyethylene bags and stored at –20 °C until further use and analysis.

The following ingredients were used in bread formulations: BSGs, Manitoba soft wheat flour type 0 (COOP, Casalecchio di Reno, Italy), water, extra-virgin olive oil (Pazienza, Foggia, Italy), gluten (Elgranero, Madrid, Spain), sodium chloride (Compagni Europea Sali, Margherita di Savoia, Italy), and dehydrated *Saccharomyces cerevisiae* yeast (Cameo, Desenzano del Garda, Italy).

### 2.3. Formulations and Bread-Making

**Figure 1:**
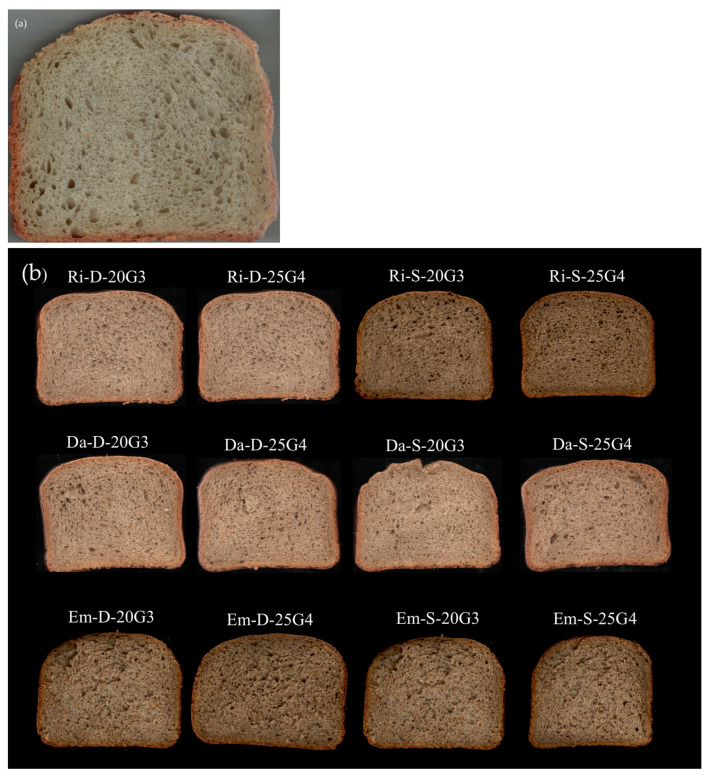
Slices of (**a**) control bread and (**b**) the 12 functional breads produced.

Thirteen types of bread were produced according to the formulations reported in Table 2: a control, made of 100% Manitoba soft wheat flour type 0 (Figure 1a); and twelve samples, obtained by replacing Manitoba flour with two different amounts (20 and 25%) of each of the six BSG flours (Figure 1b). When 20% of Manitoba flour was replaced, gluten was also added in an amount corresponding to 3% of the total weight of flours (BSG + Manitoba), while at 25% of flour substitution, a corresponding 4% of gluten was added, always referred to in the total amount of flours. Relying on the results of preliminary tests, we decided to use the same water amount for all formulations, since it was sufficient to ensure the right level of hydration and the correct development of the dough without changing the production times. For each type of breads, three technological replicates were performed.

Loaves of regular shape were produced using a mould of 20 cm length and 11 cm width. In order to standardise the production, the entire procedure was performed in a bread-making machine (Zero-Glu Pro, Imetec, Azzano S. Paolo, Italy) using the program number 14 (total duration 3 h and 12 min) that includes the following steps: kneading, 22 min; 1st leavening, 40 min; 1st stirring, 5 s; 2nd leavening, 73 min; 2nd stirring, 5 s; 3rd leavening, 50 min; cooking, 47 min.

### 2.4. Analysis of BSGs

Moisture and ash contents were determined according to the AACC Methods 44-15.02 and 08-01.01, respectively, and expressed as % [22].

BSG proteins were extracted and analysed through electrophoretic and chromatographic techniques. Total proteins from BSG samples (~1 g) were extracted for 3 h with 10 mL of a buffer containing Tris-HCl 0.0625 M (pH 6.8), SDS 2%, and glycerol 10% (*v*/*v*) and then the extracts were centrifuged at 13,000× *g* for 10 min at 25 °C. Supernatants were carefully removed and stored at −20 °C until the analysis. Protein content was determined using Quick Start™ Bradford Protein Assay (Bio-Rad, Hercules, CA, USA), according to the supplier instructions. Electrophoretic separation (SDS-PAGE) was performed through a Mini-PROTEAN Tetra system electrophoresis cell (Bio-Rad, Hercules, CA, USA), according to la Gatta et al. [23] as follows: loading of 10 μg of sample onto the gel; running buffer consisting of Tris 0.025 M, Glycine 20 mM, 1% SDS; application of a potential of 200 V for one hour; use of a Prestained SDS-PAGE standard (Bio-Rad, Richmond, CA, USA). The gels were stained with a 0.25% (*w*/*v*) solution of Coomassie Brilliant Blue G-250 (CBB), fixed with a 7% (*v*/*v*) solution of acetic acid and 40% (*v*/*v*) of methanol, and destained with water. The polymeric components of BSG proteins were analysed using a two-step extraction procedure. An aliquot of each sample (20 µL) was analysed according to la Gatta et al. [23] using a Biosep SEC-S4000 column (300 × 7.8 mm, Phenomenex, Torrence, CA, USA). The fractions of total unextractable polymeric proteins (tUPP%) and of large unextractable polymeric proteins (lUPP%) were calculated according to Kuktaite et al. [24]. In addition, the incidence of unextractable (both monomeric and polymeric forms) proteins on the total protein content was also calculated (UP%).

The extraction of total phenolics was performed according to Gandolpho et al. [25] with some modifications. Briefly, 1 g of each BSG was suspended in a hydroalcoholic solution (ethanol 58%, solid-to-liquid ratio 1 g to 30 mL) and treated in an ultrasonic bath according to the following conditions: 37 kHz, 30 °C, 30 min. After extraction, the solutions were centrifuged (2000× *g*, 25 min, 20 °C) and the supernatants were removed and filtered through nylon filter (0.45 μm). The filtered extracts were analysed for their phenolic content and antioxidant activity. TPC was determined using the Folin–Ciocalteu reagent [26] and expressed as mg of gallic acid/g of dry matter. The phenolic profile was analysed by a HPLC system equipped with a diode array detector (Agilent 1100 Liquid Chromatograph, Santa Clara, CA, USA) according to Aliakbarian et al. [27] using a 100 × 4.6 mm × 3 µm RP-C18 Gemini column (Phenomenex, Aschaffenburg, Germany). The wavelengths used were 250, 280, and 320 nm. Identification of phenolics was made taking into account their retention times and comparing their spectra with those of standard materials while their quantification (mg/g dm) was obtained by comparing their peak areas with those of standard curves. The antioxidant activity was measured using 2,2-diphenyl-1-picrylhydrazyl (DPPH) free radical scavenging [28] and results were expressed as mg of Trolox (6-hydroxy-2,5,7,8-tetramethylchroman2-carboxylic acid) per g of dry matter.

Soluble and insoluble dietary fibres were determined according to AACC Methods 32-05.01 and 32-21.01 [22] using the K-TDFR-200A Megazyme kits (Neogen Europe Ltd., Ayr, Scotland) and expressed as %.

### 2.5. Analysis of Functional Bread

Previously ground bread slices were submitted to the determination of moisture, ash, TPC, antioxidant activity, phenolic profile, and dietary fibre, and to the protein characterization as already described for BSG in Section 2.3.

In order to evaluate the chromatic and structural characteristics of the bread samples, the images of the slices were acquired with an Epson scanner (mod. XP-3100, Cinisello Balsamo, Italy) at a resolution of 1200 dpi and saved in the tiff format. A ruler was placed next to the samples in order to convert pixels to centimetres through the measurement tools of the image analysis software. The image processing was performed with ImageJ ver. 1.52a, a free public domain software developed at the National Institutes of Health (USA). The crust and crumb colours were expressed according to the coordinates of the colour space defined by the International Commission on Illumination: L*, lightness/brightness from black to white on a scale of zero to 100 and a* and b*, which represent chromaticity with no specific numeric limits. Negative and positive a* values correspond with green and red, respectively, while negative and positive b* values correspond with blue and yellow, respectively. The following structural characteristics were determined: slice height; crumb specific volume (cm^3^/g); number of pore/cm^2^; average pore size (mm^2^); porosity %, i.e., the surface of the slice occupied by pores; pore circularity, calculated as 4π*area/perimeter^2^ (it ranges between 1.0 and 0.0, with a value of 1.0 indicating a perfect circle and value near to 0.0 indicating increasingly elongated shapes).

A panel of 10 trained judges (5 females and 5 males) between 20 and 65 years of age, experienced in the sensory evaluation of baked foods, carried out a Quantitative Descriptive Analysis (QDA) in a sensory laboratory equipped with booths at 23 ± 1 °C, under a white light. All samples were assessed 2 h after baking. A slice of each bread sample was randomly labelled with a three-digit numeric code and provided to assessors in a double-blind presentation to avoid any expectation error. The attributes were selected among those found in literature and generated by the same panel so as to give an overall description of the products without overlapping. As a result of this selection, the profile sheet used by panellists for the QDA of bread samples included 5 visual (for crust: colour and thickness; for crumb: colour, pore size, and development), 6 olfactory (on crust and crumb together: overall, freshly baked bread, wheat, and malty smell; toasty smell on crust; yeast smell on crumb), 4 gustatory (sweetness, saltiness, and acidity/sourness of crumb; crust bitterness), and 6 tactile (for crust: hardness and crispiness; for crumb: resistance to chewing, cohesiveness, graininess/fibrousness, stickiness) attributes to evaluate on numeric category scales. Unipolar category scales were used for all the attributes with the exception of tactile attributes, which were evaluated on bipolar category scales. The attribute definitions were retrieved from Callejo [29] and supplied to the panellists that were also asked to evaluate the overall quality of each bread, i.e., the comprehensive likeness/dislikeness of the sample expressed considering all the sensory attributes previously evaluated [30]. The panellists rated the intensity of each parameter from 0 (minimum scale) to 9 (maximum scale). Judges were instructed to rinse their mouths between samples with natural water. To prevent sensory fatigue, the samples were divided into two subsets, one completely evaluated in the morning tasting session and another one completely evaluated in the session of the afternoon of the same day.

### 2.6. Statistical Analysis

Each analysis was replicated at least three times, with the exception of the chromatographic analyses of proteins, which were performed in duplicates, and the image analysis, with five acquisitions for each sample. The averages and the standard deviations were calculated. Analysis of Variance (ANOVA) and LSD test were applied (*p*-value < 0.01) to study the single and interactive effects of type of BSG and geographical origin (of the unmalted cereal in the mixtures used in brewing) on the characteristics of BSGs. The same statistical analyses were applied to evaluate the single and interactive effects of type of BSG, geographical origin (of the unmalted cereal in the mixtures used in brewing), and amounts of BSG-gluten used in the formulation of the characteristics of the functional breads. Principal Component Analysis (PCA) was applied to evaluate the possibility of homogeneously grouping both BSGs and breads according to the experimental data. Pearson correlation coefficients at *p*-value < 0.01 was applied to individuate significant correlations among pairs of characteristics of bread samples. The statistical analyses were carried out using Excel software V. 14.0.0 for Mac and Statistica for Windows V. 7.0. (Statsoft, Tulsa, OK, USA).

## 3. Results and Discussion

### 3.1. BSGs

#### 3.1.1. Physicochemical Characteristics of BSGs

Moisture was determined to check that drying was successful and to verify that the amount of water indicated in the bread formulations (Table 2) ensured the right dough hydration. According to the results of Table 3, neither type of BSGs nor their origin significantly affected the residual moisture. The mean moistures ranged from 2.9 to 3.5%, thus ensuring the inhibition of microbial growth [31]. In agreement with the findings of previous studies, BSG is a good source of ash with a great variability among samples depending on both the type and geographical origin of BSG [32,33]. The highest ash content was observed in Em spent grains coming from Daunia area while the lowest concentrations were detected in **Ri** samples (Table 3). The differences among BSG types depended on the use of not de-hulled emmer while the influence exerted by the geographical origin was due to the substantial differences in soil and climate conditions between Daunian sub-Apennines (D) and Salento peninsula (S), being the same agricultural practices adopted [34,35].

**Figure 2:**
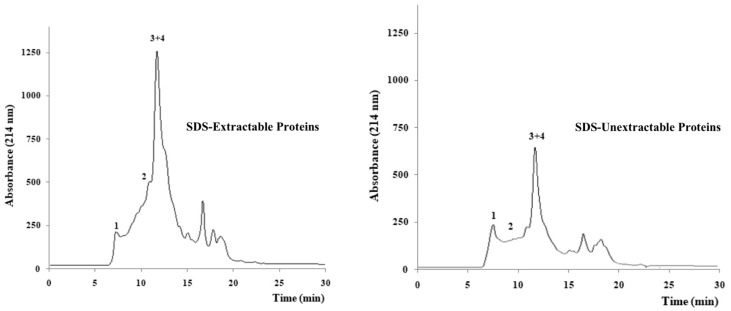
Chromatographic profiles of SDS-Extractable and SDS-Unextractable proteins of Em-D spent grains: peak 1, Large Polymeric Proteins (LPP); peak 2, Small Polymeric Proteins (SPP); peak 3 and 4, Large Monomeric and Small Monomeric Proteins (LMP + SMP).

BSG showed the common chromatographic profiles (Figure 2) of SDS-extractable and SDS-unextractable cereal proteins with the elution of four main peaks between 7 and 14 min: peak one, related to the elution of Large Polymeric Proteins (LPP), peak two related to the elution of Small Polymeric Proteins (SPP), peak three related to the elution of Large Monomeric proteins (LMP), and peak four related to the elution of Small Monomeric Proteins (SMP) [23,24]. The importance of studying the size distribution of polymeric proteins is due to its influence on the technological properties of flour [36]. Since UPP% of wheat cultivars is affected by both genetic and environment, this index has been suggested as an effective evaluation parameter in breeding programs of locally adapted wheats intended for bread-making, even considering the effects of environmental factors such as temperature, and nitrogen application and timing [37,38,39]. In agreement with the finding of a previous work [36], the proportion of the total unextractable polymeric proteins (tUPP%) in BSG samples was significantly affected by genotype and environment, whether considered individually or not. In addition, our results highlighted that the interactions of the two factors was also statistically significant, and that some considerations can be extended to the fate of the large unextractable polymeric fraction (lUPP%). The highest tUPP% and lUPP% were quantified in Em spent grains from Salento while the lowest values were found in Da spent grains from Daunia. Regarding the effects of BSG type, a previous research already demonstrated a significantly higher aggregation of emmer gluten proteins with respect to that of durum wheat, which was due to differences in the amino acid sequences between the glutenin subunits of the two cereals [23]. As can be inferred from Table 3, Da-D, Da-S, and Fa-S showed a percentage of total unextractable polymeric proteins higher than that of the large unextractable polymeric proteins, while Ri-D, Ri-S, and Fa-D showed the opposite behaviour. These results would suggest a different polymeric protein distribution between the two groups of samples, with the second group characterised by the presence of high molecular weight aggregates. These findings are in agreement with the results obtained by Johansoon et al. [40], who observed that the protein size distribution is affected by genotype and, for the same cultivar, by weather conditions. The percentage of unextractable (both monomeric and polymeric forms) proteins was also calculated. The highest and the lowest UP% were quantified in Em and Da spent grains, respectively, while any differences could be attributed to geographical origin. This independence of environment would indicate UP% as a more useful tool than total and large UPP% in screening genotypes suitable for bread-making.

During brewing most of the water-extractable and soluble compounds are released into the wort, so BSG are mainly constituted of water-unextractable or insoluble compounds (cellulose and non-cellulosic polysaccharides) and the few water-extractable and soluble compounds (feruloylated arabinoxylans with a wide range of molecular mass) are entrapped in the complex matrix made of cellulose, protein, and lignin [41]. In our work, the percentage of insoluble dietary fibre ranged between 21.58% and 34.44% and both interactive and single effects of BSG type and geographical origin were significant. The highest IDF% were detected in Em, followed by Ri, and Da. BSGs from Salento generally had higher insoluble fibres than the corresponding samples from Daunia. The interactive and single effects of the considered factors were statistically significant for soluble dietary fibres as well. The percentages of SDF were very low in all brewers’ spent grains, ranging from 0.42% to 2.06%. The lowest percentages of soluble fibres were detected in Da samples too, while Ri spent grains had the highest SDF%. The percentages of soluble fibre on the total fibre ranged from 1.89% (Da-S) to 7.04% (Ri-D). Concerning the influence of geographical origin, the soluble fibres showed a behaviour opposite to that of the insoluble fraction, with the highest percentages of SDF detected in samples from Daunia. The amounts of IDF and SDF were consistent with those found by Shih et al. [42] in BSGs from India Pale Ale brewers (27.94–48.93% for IDF; 1.44–2.10% for SDF). The total dietary fibre was between 21.99% of Da-S and 35.34% of EmS, resulting within the ranges found in the literature (28.22–42.6%) [43,44]. The highest IDF% and TDF% of Em spent grains strongly depended on the use of the non-dehulled emmer while the differences between Ri and Da were determined by genotype [45].

#### 3.1.2. Phenolic Concentration and Antioxidant Activity of BSGs

The interest towards phenolic content and the profile of BSG is due to the bioactive and antioxidant effects of these compounds and to their contribution, together with fibres, to the functional effects of bread and other cereal derivatives obtained with a partial replacement of wheat flour with nutraceutical ingredients. Nevertheless, it must be pointed out there is still no scientific evidence to support health claims concerning the phenolic compounds of cereals, as a consequence of the co-occurrence of other bioactive compounds (just like fibre in wholegrain products) that hinders verification of any health benefits exerted by these compounds [46]. During the mashing and sparging steps, phenolic compounds are released from the starting cereal mixtures (where they accumulate mainly in bran fractions) to wort but significant amounts remain in the resulting BSG, mainly bound to cell walls as polysaccharides or proteins but also, in smaller amounts, as free compounds. The mean total phenolic content of BSG, comprised between 3.114 and 4.868 mg/g d.m. was significantly influenced by the type of BSG with the highest and the lowest phenolic contents detected in Em and Ri spent grains, respectively. The effects of the place of origin were not statistically significant (Table 4). Since the six starting cereal mixtures were formulated by adding the same barley malt to a constant amount (35%) of each unmalted cereal, the influence exerted by the type of BSG on its phenolic content must be unquestionably attributed to the species and variety of the unmalted grains [47] or, in other words, to their specific phenolic profile and the distribution of free, conjugated, and bond forms. This idea is reinforced by the finding that different phenolic compounds are released in different amounts during mashing. In fact, according to Langos and Granvogl [48], ferulic and *p*-coumaric acids are released in higher extension (up to 9-fold) compared with cinnamic acid and, as inferred by Schwarz et al. [49], the optimal pH ranges for the extraction of ferulic acid, *p*-coumaric acid, and cinnamic acid are different, being 5.4–6.6, 5.8–6.0, and 6.8, respectively.

The comparison of our results with the findings of other researchers is not simple since the latter generally concerns BSGs deriving from a mixture of various malts (instead of mixtures of malted and unmalted grains as in our work) or spent grains of unknown origin and nature [15,50]. Nevertheless, TPC of our BSG samples were within the range of concentrations found by Birsan et al. [51] in light and darker BSGs (from 3.01 ± 0.19 to 4.71 ± 0.28 mg/g d.m.) but much greater than those detected by Farcas et al. [20] in 100% malt BSGs (from 0.36 to 2.79 mg/g d.m.).

The mean antioxidant activity, ranging from 0.64 mgTrolox/g d.m. of Em-D to 2.89 mgTrolox/g d.m. of Ri-S, was significantly influenced by the type of BSG with the highest value measured on **Ri**. The effects of the place of origin were not statistically significant (Table 4). Our data confirmed that TPC and antioxidant activity were poorly correlated (R^2^ 0.4997, *p* < 0.01); regardless, phenolic compounds are the most important group of antioxidants [52].

Regarding the phenolic profiles (Table 4), eleven compounds were detected in all the BSGs: six phenolic acids (gallic, 4-hydroxybenzoic, vanillic, caffeic, ferulic, *p*-coumaric); two flavanols (catechin and epicatechin); two flavonols (quercetin and kaempferol); and one hydroxystilbene (resveratrol). Kaempferol (0.795–1.018 mg/g d.m.), epicatechin (0.160–0.168 mg/g d.m.), and caffeic acid (0.014–0.178 mg/g d.m.) were the main phenolics. The other compounds had concentrations always lower than 0.050 mg/g d.m. Regarding the single effects exerted by the type of BSGs on their phenolic profiles, Em showed the highest amounts of all phenolic compounds except gallic acid, detected in higher concentrations in Ri, and vanillic acid, whose concentrations was the same in all BSGs. The single effects of geographical origin was not statistically significant for vanillic, caffeic, and *p*-coumaric acids. The highest amounts of other compounds were retrieved in BSGs from Daunia.

### 3.2. Functional Breads

#### 3.2.1. Physicochemical Characteristics of the Functional Breads

Colour, moisture, ash, and the protein distribution of breads enriched with BSGs are reported in Table 5 and compared with the characteristics of the control bread.

Regarding colour indices, the crust of the control bread showed the highest L* (together with Em-D breads) and b* (with Da-S-20G3) values and the lowest a* indices (with Da-D-25G4, Da-S-20G3, and Em-D-20G3). Em-S-25G4 had the lowest L* and b* values, while the highest a* was measured on Da-S-25G4. The crumb of the control bread also showed the highest L* and b* (with Ri-D-20G3) values and the lowest a* indices.

The type of BSG exerted significant effects on all colour indices. Concerning crust, the highest brightness and yellow index were measured on Da samples and the lowest red index was detected in Ri breads. The crumb of Ri breads was the darkest, the reddest (with Da samples), and showed the lowest yellow index (together with Em). The different behaviour observed for the various types of BSG was correlated to the colour of the starting spent grains.

The level of BSG-gluten addition significantly affected the colorimetric indices of bread with the exception of the crust a* values, but the effects were opposite between crust and crumb. The increase in BSG-gluten content generally decreased the L* and b* values of the crust, i.e., determined the formation of a darker colour. This behaviour is explained by the higher protein content in the formulation which caused intensive Maillard reactions [53]. Contrary to what happened to the crust, the increase in BSG-gluten content corresponded to a slightly lighter crumb (due to the clear colour of the fibre added) but also to a more intense golden colour (increased a* and b* values). In most previous works, a darker crumb was observed as a consequence of BSG addition [15] but Gómez et al. [54] pointed out that the effects depend on the colour of fibres contained in the added ingredient.

Remarkable water content was quantified in the control bread. However, other bread samples showed higher crust moisture (Em-D-20G3) and similar amounts of water in crumb (Em-D-25G4). The type of BSG did not affect crust moisture while the crumb of breads produced with Da spent grains contained generally higher water in their composition. The crumb moisture was significantly and positively affected by the BSG-gluten level, and this behaviour may be attributed to the higher fibre and protein contribution [17].

Regarding ash, the lowest and the highest amounts were quantified in control (3.1%) and Em-S-25G4 (3.6%) breads, respectively. The addition of all the BSG types significantly increased bread ash content but, consistently with the BSG composition reported in Table 3, the greatest ash amount was contributed by the Em type. Since BSG is mainly composed of the husk of grain and minerals are present in a greater amount in their outer layer, the amount of BSG-gluten added also had a significant and positive effect on the bread ash content [17,55].

Concerning protein size distribution in bread samples, the chromatographic profiles highlighted the presence of only two main peaks, eluted between 10 and 12.5 min (i.e., the range of large and small monomeric proteins) and the total lack of peaks in the elution range of polymeric protein aggregates. This result could be due to a variety of factors including technological process; BSG diluting effect on the protein network; and possible accumulation of low-molecular weight metabolites (mainly glutathione) deriving from the lysed yeast cell. Glutathione (GSH) was found to be responsible for the chemical modification of the gluten protein structure, leading to its depolymerization [56] and the formation of lower molecular weight gluten proteins [57,58]. Consequently to these changes, unextractable proteins included only monomeric forms (Table 5). From the comparison of data in Table 3 and Table 5, a remarkable increase in UP% from BSGs to the corresponding breads can be inferred. It could be due to baking, whose high temperatures are known to induce interactions among different class of proteins, thus leading to the formation of larger aggregates or a supramolecular structure. The average unextractable monomeric proteins ranged from a minimum of 35.91% in Em-S-20G3 to a maximum of 42.07% in Ri-S-20G3. Percentages higher than 40% were also detected in Ri-D-25G4, Ri-S-25G4, and the control bread. Significant single effects of BSG type were observed, with UP% higher in Ri breads and lower in Em breads.

The geographical origin of the starting cereal mixtures did not significantly influence any of the parameters of Table 5.

#### 3.2.2. Phenolic Concentration, Antioxidant Activity, and Fibre Content of the Functional Breads

The amounts of antioxidants and fibres are among the most important factors to consider in assessing the nutraceutical quality of breads.

The total phenolic contents of functional breads were always higher than those of control bread (1.555 ± 0.158 mg/g d.m.) and comprised between 1.793 ± 0.183 mg/g d.m. (Ri-D-20G3) and 2.833 ± 0.772 mg/g d.m. (Da-S-25G4) (Table 6). These were the results of the interactions among the type of BSG, the geographical origin of cereal mixtures, and the amount of BSG-gluten, although the single effects of these variables were also statistically significant (with the exception of the geographical origin). In particular, the highest TPCs were detected in Em breads, consistently with the high phenolic concentrations of the starting spent grains. Moreover, the bread antioxidant content increased with the increase in BSG-gluten amount in the bread formulation. Finally, it is appropriate to consider that the phenolic content of the final breads was also affected by bread-making and, although the same process was applied for all samples, the magnitude of these changes could not be the same. According to a recent research of Tian et al. [59], bread-making generally improved the potential health benefits of whole wheat products. The authors pointed out that fermentation and baking generally increased soluble phenolic content and its antioxidant activity due to the contribution of Maillard reaction products, and that those steps only slightly increased the insoluble phenolic fraction and its antioxidant activity. The TPCs of our experimental functional breads were considerable higher than those (0.47 ± 0.06 mg/g d.m.) retrieved in the recent literature [60].

The antioxidant activity values of the functional breads showed trends similar to those of TPCs. They were always higher than those of the control bread (0.36 ± 0.02 mg Trolox/g d.m.) and comprised between 0.67 ± 0.16 mg/g d.m. (Ri-D-20G3) and 3.45 ± 0.47 mg/g d.m. (Em-D-25G4) (Table 6). The single effects of the type of BSG and the amount of BSG-gluten were statistically significant. In particular, the highest and the lowest antioxidant activity values were detected in breads produced with Em and Ri spent grains, respectively. Furthermore, the bread antioxidant activity increased with the increase in BSG-gluten amount in the bread formulation. TPC and antioxidant activity showed a strong correlation (R = 0.885, *p*-value < 0.01).

The bread phenolic profiles were simpler than those of the starting BSGs. Five phenolic compounds were identified in all the functional breads, including four phenolic acids (gallic, vanillic, caffeic, and sinapic) and a flavanol (epicagallotechin gallate). Nevertheless, their concentrations depended on BSG type, the geographical origin of the stating cereal mixtures, and BSG-gluten amount. *p*-Coumaric acid was detected only in the control bread that, instead, did not contain phenolics such as epigallocatechin gallate, and vanillic, caffeic, and sinapic acids. The interactive effects of the three factors were also statistically significant. The type of BSG showed significant single effects, with the highest concentrations of almost all compounds detected in Em breads. The higher level of BSG-gluten supplementation allowed to obtain breads with a higher content of almost all compounds. The single effects of the geographical origin of the starting mixtures were less significant.

The interest towards the fibre content of BSG-enriched bread is due to the possibility, established by Reg. (EU) N°. 432/2012 [61], to use the following two health claims: “Barley grain fibre contributes to an increase in faecal bulk” for foods which are high in that fibre, i.e., for those foods that contain at least 6 g of fibre per 100 g or at least 3 g of fibre per 100 kcal; and “Beta-glucans contribute to the maintenance of normal blood cholesterol levels” for food which contains at least 1 g of beta-glucans from barley/barley bran per quantified portion. [62]. The increase of faecal bulk is related to the ingestion of insoluble dietary fibre. Other long-known benefits of dietary fibre intake include the modulation of glycaemic index and potential prebiotic capacity, which are known to be linked to arabinoxylan and arabinoxylan-oligosaccharides, the latter primarily deriving from wheat [63,64]. USDA recommends daily intakes of fibres equal to 25 g for women and 38 g for men up to 50 years old and to 21 and 30 g for elder women and men, respectively [65]. Since the actual intake is generally lower, especially in Western countries, the regular consumption of BSG-enriched breads could help consumers to meet such recommendations. As can be inferred from Table 6, the amounts of IDF and SDF in BSG- enriched breads were significantly lower than in the corresponding spent grains, due to the dilution effect of Manitoba flour. IDF% and SDF% felt both interactive and single effects of the factors. More in depth, IDF% ranged from 2.05% in the control bread to 7.15% of Em-S-25G4 while SDF% was in the 0.004% (Ri-S-25G4)–1.56% (Da-S-25G4) range and the percentages of soluble fibre on the total fibre were appreciably higher than in the BSG samples, ranging from 0.06% (RiS25-G4) to 22.51% (DaS25-G4), thus contributing beneficial effects that go beyond the simple increase in faecal bulk. A first reason for this behaviour is the high percentages of soluble fibre in the Manitoba flour. In fact, the control bread had a percentage of soluble fibre on the total fibres equal to 23.49%. Another reason is that, during bread-making, a decomposition of dietary fibre (first hemicellulose and afterwards cellulose) occurred, reducing the fibre molecular weight [66]. The reason for this degrading action could be both the yeast, since some *Saccharomyces cerevisiae* strains are able to produce cellulase and xylanase [66], and the first step of the baking process when both temperatures and moisture are elevated, thus simulating the conditions of an autoclave treatment [67]. In the control bread, the incidence of soluble fibre on the total amount of fibre was equal to 23.49%. However, while data indicate that the single effects exerted by BSG type and the geographical origin on bread IDF% were similar to those observed for spent grains, they also describe the opposite effects of BSG type and geographical origin on the soluble fibre fraction between breads and spent grains. Significant increases in both IDF% and SDF% were evaluated by increasing the amount of BSG-gluten added. The total dietary fibres ranged between 4.50% of RiD20-G3 and 7.85% of EmS25-G4, resulting as significantly higher than those quantified in the control bread (2.68%) and slightly higher than the ranges found in recent literature (3.32–6.37%) [44]. According to the data concerning the dietary fibre contents of the enriched breads, the inclusion of Em spent grains in percentages equal or higher than those used in these experiments could allow the use of one or both of the health claims mentioned above [61].

#### 3.2.3. Structural Characteristics of the Functional Breads

Consumers are more likely to purchase well-leavened and regularly shaped breads. For this reason, height and specific volume are often considered as the key quality parameters.

In our work, the minimum and maximum slice heights were used as indices of bread shape regularity. The highest values of the minimum (9.36 ± 0.24 cm) and maximum (10.14 ± 0.17 cm) height were measured on the control bread, while the lowest values were found for Da-S-20G3 (7.72 ± 0.84 cm) and Ri-D-25G4 (8.39 ± 0.20 cm), respectively (Table 7). As can be inferred from these data, slice height values were significantly affected by interactions among BSG type, origin, and the amount of BSG-gluten, but the BSG type also exerted significant single effects, with the highest values observed in Em breads. The crumb specific volume is another key parameter since superior bread quality is often characterised by high specific volumes [44]. Crumb specific volume (Table 7) was only slightly reduced by the BSG addition, ranging from 2.11 ± 0.9 cm^3^/g of Da-D-20G3 to 2.64 cm^3^/g of Em-D-25G4 and control bread. These results were significantly better than those observed by Amoriello et al. [15] in breads produced with medium or strong wheat flour supplemented with 5 or 10% of BSG. In that work, the authors attributed the limited dough development of BSG-enriched breads to a reduction of extensibility and the gas-retention ability of gluten, in turn caused by dilution with non-gluten proteins and disruption due to the interference of fibres. The better specific volume of supplemented breads obtained in our work was determined by the concurrent addition of gluten. As always in our work, only the interactive effects of the three factors were significant, but it is interesting to point out that, although not significant, the highest specific volumes were quantified in Em breads, especially at increasing amounts of BSG-gluten added, i.e., in the samples with the highest fibre contents and deriving from the spent grains that had the highest amounts of total and large unextractable polymeric proteins.

Pores are created within the dough structure as a consequence of CO_2_ production during leavening. Their characteristics are described in Table 7. The control bread had a high pore density (0.73 ± 0.02 pores/cm^2^) but not the highest, since that index ranged from ~0.3 pores/cm^2^ (Ri-S breads, Em-D-25G4, and Em-S-25G4) to ~0.82 pores/cm^2^ (Em-D-20G3). This result was not in agreement with the findings of Neylon et al. [44], who observed a significant decrease in the number of cells in BSG-fortified breads. BSG type, origin, and the amount of BSG-gluten exerted statistically significant single and interactive effects, with the higher density measured on breads produced with Da-D spent grains at the lowest level of addition. The average pore size was inversely correlated (R = 0.938, *p*-value < 0.01) with pore density, ranging from 0.10 mm^2^ (control bread and Da-S-25G4) to 0.30 mm^2^ (Em-D-25G4 and Ri-S-20G3). As for pore density, the results were not in agreement with the findings of Neylon et al. [44], who observed a decrease in the cell diameter in BSG-fortified breads. Only the BSG type showed a significant effect on pore size, with the lowest values observed in Da breads. Porosity % ranged from ~35% (control bread, Da-D-20G3, and Ri-D-25G4) to ~47% (Em-S-25G4) and, as for the average pore size, only the BSG type showed a significant effect, with the highest values observed in Em breads. According to these results, the crumb of Em breads had a less compact structure than those of all other breads. Porosity % was well correlated with the total pore surface (pore density × verage pore size), showing an R value of 0.694 (*p*-value < 0.01). Circularity ranged from 0.774 ± 0.029 (Ri-S-20G3) to 0.829 ± 0.029 (Em-S-25G4), thus indicating a predominantly circular shape. Nevertheless, data showed a remarkable variability already within the samples of each type of bread and only the interactive effects of the three factors were statistically significant, making it difficult to understand the weight of each independent variable.

#### 3.2.4. Sensorial Characteristics of the Functional Breads

Control bread and breads enriched with the BSGs were evaluated by a trained panel through a Quantitative Descriptive Analysis and the results are reported in Table 8. Two of the characteristics that judges were requested to evaluate, namely crust bitterness and crumb stickiness, were not detected for any of the experimental breads. The interactive effects of BSG type, the origin of cereal mixtures, and the amount of BSG-gluten added were significant for all the other sensorial parameters.

Bread colour is the first variable evaluated by consumers and strongly affects their willingness to purchase and the product acceptability. In agreement with the findings of Ginindza et al. [68], the addition of BSG always resulted in a significant colour change compared with the control bread and the reason is that BSG contributed a higher amount of aminoacids, thus favouring the non-enzymatic browning reactions [69]. The Em breads were evaluated as the darkest. The single effects of geographical origin and the amount of BSG-gluten added were not significant either for crust or for crumb colour. Regarding the other visual characteristics, the type of BSG had significant single effects on crust thickness (higher in Da breads while control bread had intermediate scores), crumb pore size (higher in Ri breads while control bread had intermediate scores), and crumb development (higher in Em samples among the BSG-enriched breads but lower than that of the control bread).

The fortified breads generally showed the highest intensity of wheat, malt, and yeast flavours. According to Ktenioudaki et al. [16], these flavours were due to volatile compounds already present in BSG (2-heptane, butanal, 2-methylbutanale, benzene, and 2,3-butanedione) and arising from fermentation (ethanol, butanol, and other acholic compounds) and Maillard reactions (furfural, pyrazine). The type of BSG exerted significant effects on overall intensity, malty, and toasty flavours (lower in Em breads), and on wheat and yeast smells (higher in Da breads). The amount of BSG-gluten added exerted significant single effects only on wheat and yeast smells, which increased with the level of replacement.

Concerning crumb taste, the single effects of the three factors were not statistically significant. The saltiness and acidity/sourness of the functional breads were evaluated as lower or equal to those of the control bread. The latter obtained intermediate scores for sweetness, perhaps as a consequence of the maltose and glucose contained in BSG. Moreover, the sweet taste was also enhanced by the volatile compounds responsible for sweet and malty flavours.

Regarding bread texture, the addition of BSG-gluten always increased resistance to chewing and the fibrousness of the functional breads with respect to the control. The control bread obtained intermediate scores for crispness. The highest crust hardness and resistance to chewing and the lowest cohesiveness and fibrousness were attributed to breads fortified with Da spent grains. The amount of BSG-gluten added significantly affected the bread texture, except for crumb cohesiveness. The hardness of the crust and the resistance to chewing of crumb increased with BSG-gluten content. This behaviour could be explained by both the higher fibre and protein contents of functional breads, which caused a greater water absorption [17,70], and also the presence of pentopans, a fibre BSG component, responsible for the gluten protein cross linking [71]. Crust crispness decreased with the increase in BSG-gluten content due to the increase in water absorbed, while crumb cohesiveness and fibrousness did not show significant changes. These results were only partially in agreement with those of Yitayew et al. [17], who found that the hardness and breakage of crumb increased with BSG level.

Finally, the best and the worse overall ratings were attributed to Ri (together with control) and Em breads, respectively, and the panellists accorded their preferences to breads produced with the lowest amount of BSG-gluten added only for DaS and Em breads. In the other case, the % of BSG added did not affect the overall sensory quality. This is an interesting finding since the percentages of BSG in our products (20 or 25%) was much higher than those (≤10%) indicated as optimal by Yitayew et al. [17] and Ginindza et al. [68] who found the maximum and minimum acceptability score for the control sample and the bread supplemented with the highest amount of BSG, respectively. The interest of our finding is also due to the fact that, in other research, the BSG-fortified foods exerted a higher appeal [42] than the not-enriched counterparts as a consequence of consumer interest for health issues, characteristics that overshadows the product sensory properties [72].

#### 3.2.5. Principal Component Analysis and Pearson Coefficients

**Figure 3:**
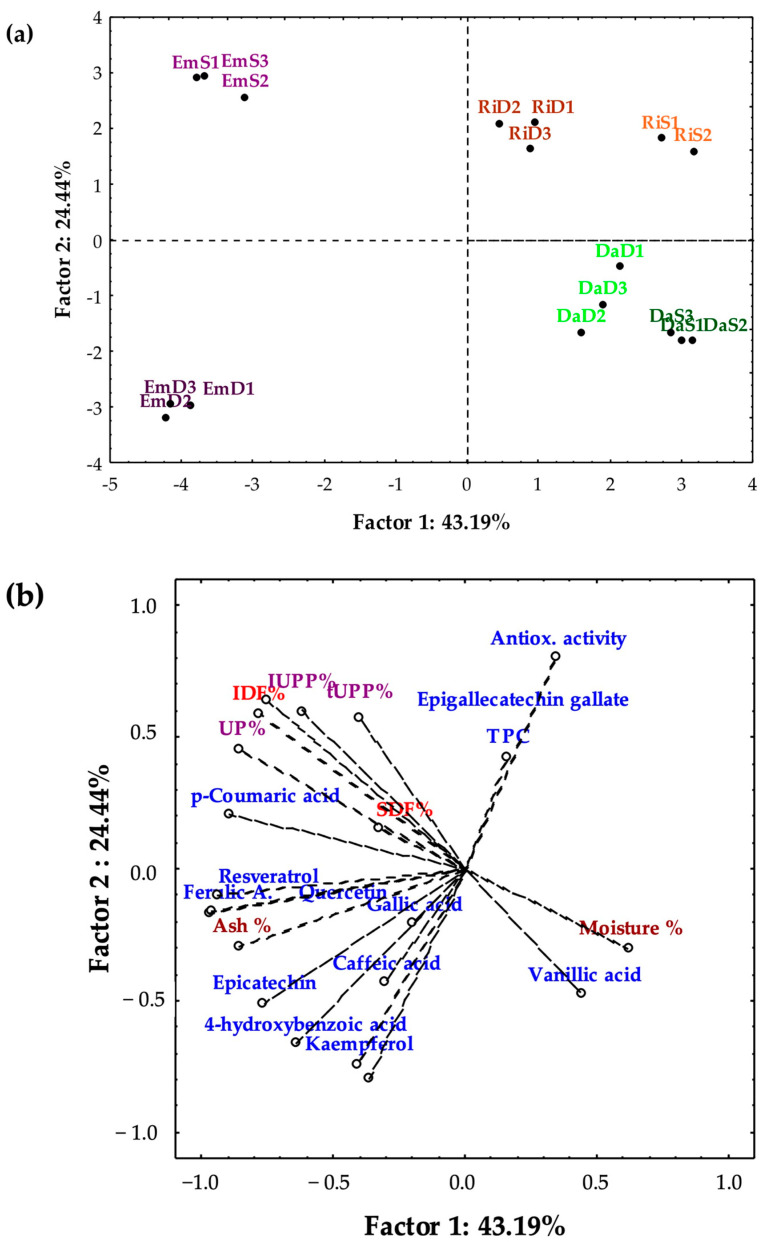
Projections of (**a**) BSG samples and (**b**) the related experimental data on the factorial plane. Phenolics are in blue font; Insoluble and Soluble Dietary Fibres are in red; tUPP%, lUPP%, and UP% are in purple; Moisture % and Ash % are in brown.

Principal Component Analysis was carried out to verify the possibility of grouping BSGs by type and/or by place of origin on the basis of their physical characteristics and the content of antioxidants, fibres, and non-extractable proteins. The ability of PCA to group BSG samples belonging to the same base malts group with a high percentage of explained variance (75%) was already highlighted by di Matteo et al. [73]. Figure 3 shows a biplot of Factors 1 and 2 that accounted for 67.63% of the variance in the whole data set. Regarding the projection of the BSG samples on the factorial plan (Figure 3a), Em samples differed from the others for their negative loading of Factor 1, accounting for 43.19% of the explained variance. Em samples were furtherly divided into two geographically homogeneous groups, one including spent grains from Daunia and another including spent grains from Salento, characterised by the negative and positive loading of Factor 2, respectively. The negative loadings of this factor were associated with high amounts of ash and most of the phenolic compounds while the positive loadings of these factors were associated with high amounts of insoluble fibres, unextractable proteins, *p*-coumaric acid, and epigallocatechingallate (Figure 3b). Ri-D and Ri-S spent grains were homogeneously grouped but close to each other in the upper right quadrant, the first with loadings near to 0, the latter with more positive loadings of Factor 1 (Figure 3a), characterised by high antioxidant activity values and low phenolic concentrations (Figure 2b). Da-D and Da-S spent grains were homogeneously grouped but close to each other in the lower right quadrant (Figure 3a). This quadrant was associated with low-to-intermediate values of all variables (Figure 3b).

**Figure 4:**
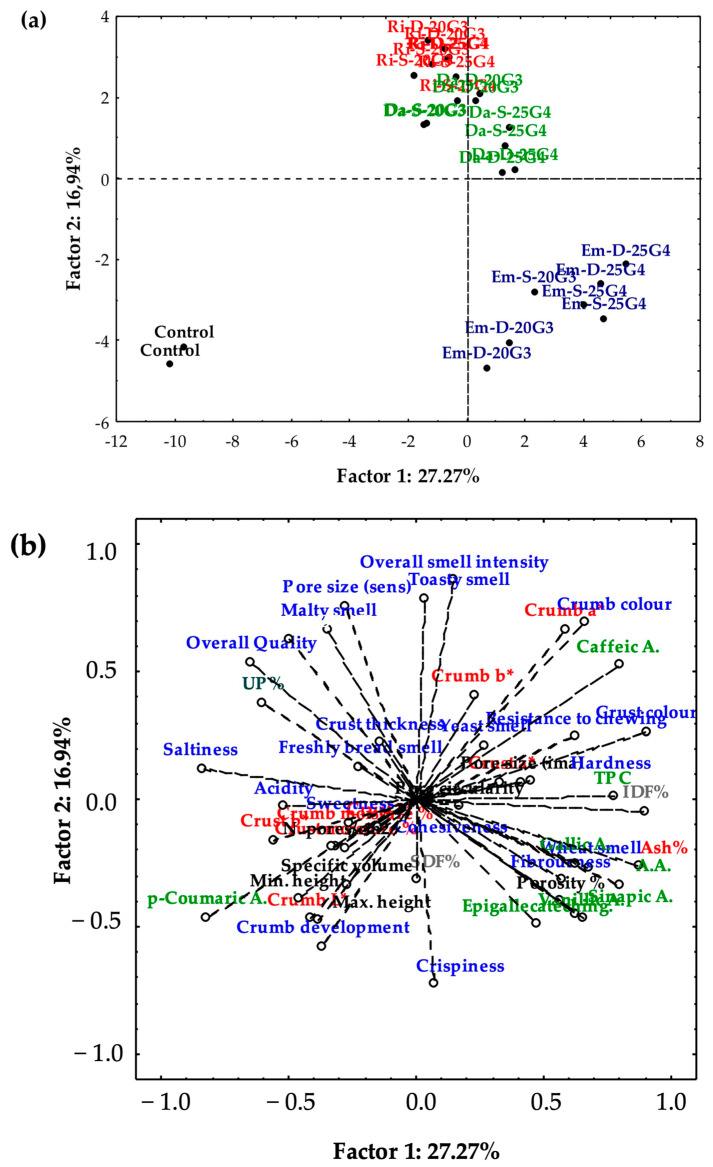
Projections of (**a**) control and functional breads and (**b**) the related experimental data on the factorial plane. Phenolics are in green font; Insoluble and Soluble Dietary Fibres are in purple; UP% is in dark green; Moisture %, Ash %, and colorimetric indices are in red; structural parameters are in black.

Principal Component Analysis was also performed to highlight the relationship between bread samples and their chemical, physical, structural, and sensory characteristics. Figure 4a,b shows the projection on the factorial plan of breads and analytical information, respectively. Factors 1 and 2 only explained 44.21% of the variance in the whole data set and made it possible to group the thirteen types of samples into just three sets that stood out for their position in the factorial plan: one including the control bread, placed in the lower left quadrant and associated with high values of crumb development, specific volume, minimum and maximum height, cohesiveness, and amount of *p*-coumaric acid; another group, comprising all the Em breads distributed within the lower right quadrant and characterised by high concentrations of IDF, TPC, gallic acid, vanillic acid, sinapic acid, epigallocatechingallate, and high values of crispiness, porosity, fibrousness, and wheat smell; and the last group, including Ri and Da breads, which were partially overlapped and concentrated straddling the two upper quadrants, associated with high values of overall smell intensity, a toasty smell, pore size, crust thickness, overall quality, a high concentration of caffeic acid, a darker crumb colour, and intermediate TFC. As can be inferred from Figure 4a, PCA of the overall data set was not able to discriminate bread samples on the base of the two different percentages of the BSG-gluten used in bread-making. In the paper of Ktenioudaki et al. [16], PCA analysis of volatile compounds was able to clear separate snack samples in homogeneous clusters for the percentage of BSG added but it is appropriate to point out that they worked with a single type of BSG instead of three types as we did.

Pearson correlation coefficients were calculated to individuate correlations among the quality characteristics of breads and measured variables. For the sake of synthesis, only the main statistically significant correlations (*p* < 0.01) are discussed.

The percentage of unextractable proteins was positively correlated with the crumb pore size (+0.61) and negatively correlated with porosity (−0.56), sensory crust hardness (−0.53), and antioxidant activity (−0.60). A possible explanation of this behaviour is that the formation of larger protein aggregates made the crumb structure more compact and entrapped phenolic compounds through protein-polyphenol complexation while increasing the water holding capacity of the crust structure.

The insoluble dietary fibre content was positively correlated with ash content (+0.83), porosity (+0.47), and crumb fibrousness (+0.51) while the soluble dietary fibre content was positively correlated with crust thickness (+0.51) and crumb sweetness (+0.70) as a consequence of the depolymerization of hemicellulose and cellulose and the production of simple sugars. To confirm the significant effects of the addition of BSGs in terms of colour changes, IDF% resulted in a significant correlation with crust b* (−0.54), crumb a* (+0.58), and the colour of the crust (+0.79) and crumb (+0.62). However, contrary to what Ginindza et al. [68] highlighted, the darkening of the colour did not negatively affect the overall quality of the bread. IDF% as an index of the amount of BSG added also affected bread taste by reducing saltiness (−0.82) and olfactory characteristics, and enhancing the intensity of the wheat (+0.80) and yeast (+0.58) smell. Contrary to what Ginindza et al. [68] highlighted, the flavour changes caused by the addition of BSG were not correlated with the overall sensory quality of bread.

TPC was positively correlated with ash content (+0.66), IDF% (+0.74), antioxidant activity (+0.70), and with the individual concentration of most of the phenolic compounds, thus demonstrating that wholegrain derivatives can be considered good sources of phenolic antioxidants.

Further significant correlations were found between the physical, structural, and sensory characteristics of breads, as in the following cases: crumb specific volume and crumb development (+0.43); porosity and crumb pore size (−0.48); crust b* and crust colour (−0.51); and crumb L*, a*, and b* and crumb colour (−0.38, +0.68, +0.43).

Finally, the overall quality of breads was positively correlated with a malty smell (0.68) and crumb saltiness (0.70), and negatively correlated with ash and IDF% (−0.72 and −0.52), and with crumb fibrousness and porosity (−0.56 and −0.58). Our results were only partially in agreement with the findings of Ktenioudaki et al. [16], who found that taste and texture were the attributes that mostly affected the overall acceptability, and with the results of Combest and Warren [74], who found significant correlations only between taste and overall liking. In fact, in our study, some smell, taste, and texture attributes are correlated with the overall sensory quality of breads.

## 4. Conclusions

The partial replacement of wheat flour with BSG resulted in significant increases in phenolic content, and insoluble and soluble dietary fibres of the enriched breads with respect to the control thus confirming the nutraceutical and functional nature of BSGs and BSG-enriched breads, respectively. Concerning BSG samples, single and interactive effects of the type and the geographical origin of the starting cereal mixtures were highlighted, to point out that their composition is mainly determined by genetics but can be significantly influenced by environmental conditions.

The highest amounts of phenolic compounds were detected in Em spent grains, followed by Da, and Ri. Em spent grains also showed the highest concentrations of both insoluble and soluble dietary fibres, followed by Ri, and Da. The supplementation with the highest percentage of BSGs exerted a positive influence on the contents of phenolics and dietary fibres without worsening the structural and sensory attributes. These results were probably due to the simultaneous addition of gluten in amounts able to counterbalance the negative effects exerted by the fibres added on the gluten network. Moreover, the increase in soluble fibres from BSGs to the corresponding breads may be partially responsible for the good structural and sensorial performance of the enriched breads compared with the control bread.

The breads enriched with Em spent grains showed the highest percentage of phenolics and insoluble fibres and are therefore the best breads in terms of the content of nutraceuticals. However, since these desirable characteristics were accompanied by a worsening of the overall sensory quality evaluated by a trained panel, the production of bread supplemented with Ri or Da spent grains represented the optimal choice, since their intermediate phenolic and fibre contents and their overall quality scores were comparable to that of the control bread.

As a practical application, the large amount of BSG produced annually in the world makes the transformation of breweries into biorefineries capable of turning this by-product into high-value, low-perishable ingredients for food and feed industries economically convenient. Since BSG composition depends on their type and origin, they should be offered for sale with labels showing the precise information on their composition, nutritional value, and the suggested percentages of use in the formulation of the finished products. On the other hand, food companies should explore the huge variety of uses of BSG, in particular to a) increase the production of bread and other food commodities to meet the needs of the growing world population, and b) study food formulations that can be labelled with health claims.

## Figures and Tables

**Table 1:** BSG composition (%) and geographical origin.

BSGs	Origin: Daunia			Barley Malt cv. Fortuna	Origin: Salento		
	Soft Wheat cv. Risciola	Durum Wheat cv. Dauno III	Emmer		Soft Wheat cv. Risciola	Durum Wheat cv. Dauno III	Emmer
Ri-D	35			65			
Ri-S				65	35		
Da-D		35		65			
Da-S				65		35	
Em-D			35	65			
Em-S				65			35

**Table 2:** Ingredients (g) used in bread formulations.

Bread Samples	BSG Flour ^1^	Gluten ^2^	Manitoba Flour (g)	Water	EVOO	NaCl	Dehydrated *Saccharomyces cerevisiae* Yeast
Control	-	-	500	350	40	12	7
Ri-D-20G3	100 (20%)	15 (3%)	385	350	40	12	7
Ri-D-25G4	125 (25%)	20 (4%)	355	350	40	12	7
Ri-S-20G3	100 (20%)	15 (3%)	385	350	40	12	7
Ri-S-25G4	125 (25%)	20 (4%)	355	350	40	12	7
Da-D-20G3	100 (20%)	15 (3%)	385	350	40	12	7
Da-D-25G4	125 (25%)	20 (4%)	355	350	40	12	7
Da-S-20G3	100 (20%)	15 (3%)	385	350	40	12	7
Da-S-25G4	125 (25%)	20 (4%)	355	350	40	12	7
Em-D-20G3	100 (20%)	15 (3%)	385	350	40	12	7
Em-D-25G4	125 (25%)	20 (4%)	355	350	40	12	7
Em-S-20G3	100 (20%)	15 (3%)	385	350	40	12	7
Em-S-25G4	125 (25%)	20 (4%)	355	350	40	12	7

^1^ The percentage of replacement of Manitoba flour with BSG is reported in brackets. ^2^ The percentage of gluten reported in brackets is referred to the total amount of flours (Manitoba + BSG).

**Table 3:** Single and interactive effects of type of BSG and geographical origin of the cereal mixtures used in brewing on physical and chemical characteristics of BSGs.

BSGs	Moisture (%)	Ash (%)	% tUPP	% IUPP	UP%	IDF%	SDF%
	Interactive effects (Type of BSG* Geographical Origin of Cereal Mixtures)						
Ri-D	3.3 ± 0.7 ^a^	3.0 ± 0.0 ^a^	34.54 ± 1.30 ^bc^	52.54 ± 3.45 ^c^	28.23 ± 0.77 ^cd^	25.25 ± 0.05 ^c^	2.06 ^f^
Ri-S	3.4 ± 0.0 ^a^	3.1 ± 0.0 ^a^	36.97 ± 2.24 ^c^	42.13 ± 5.16 ^b^	25.33 ± 1.30 ^ab^	27.25 ^c^	0.64 ^b^
Da-D	3.3 ± 0.3 ^a^	4.8 ± 0.3 ^b^	28.82 ± 0.21 ^a^	25.96 ± 0.46 ^a^	26.05 ± 0.55 ^bc^	23.97 ^b^	1.04 ^d^
Da-S	3.5 ± 0.4 ^a^	2.9 ± 0.0 ^a^	30.45 ± 0.39 ^ab^	20.91 ± 0.46 ^a^	23.30 ± 0.01 ^a^	21.58 ^a^	0.42 ^a^
Em-D	3.0 ± 0.5 ^a^	6.7 ± 0.4 ^d^	37.07 ± 1.03 ^c^	49.96 ± 0.50 ^bc^	28.79 ± 0.35 ^d^	28.39 ^d^	1.43 ^e^
Em-S	2.9 ± 0.4 ^a^	5.6 ± 0.5 c	90.82 ± 0.22 ^d^	87.65 ± 1.40 ^d^	32.23 ± 0.33 ^e^	34.44 ^e^	0.90 ^c^
Significance	n.s.	*	*	*	*	*	*
	Single Effect (Type of BSG)						
Ri	3.3 ^a^	3.1 ^a^	35.76 ^b^	23.43 ^b^	26.78 ^b^	27.25 ^b^	1.35 ^c^
Da	3.4 ^a^	3.9 ^b^	29.64 ^a^	47.33 ^a^	24.67 ^a^	22.78 ^a^	0.73 ^a^
Em	2,9 ^a^	6.2 ^c^	63.94 ^c^	68.80 ^c^	30.51 ^c^	31.41 ^c^	1.17 ^b^
Significance	n.s.	*	*	*	*	*	*
	Single Effect (Geographical Origin of Cereal Mixtures)						
D	3.2 ^a^	4.8 ^b^	33.48 ^a^	42.82 ^a^	27.69 ^a^	26.54 ^a^	1.51 ^b^
S	3.3 ^a^	3.9 ^a^	52.75 ^b^	50.23 ^b^	26.95 ^a^	27.76 ^b^	0.65 ^a^
Significance	n.s.	*	*	*	n.s.	*	*

In column, different letters indicate significant differences at *p* < 0.01; * *p* < 0.01; n.s.: not significant.

**Table 4:** Effects of type of BSG and geographical origin of the cereal mixtures used in brewing on phenolic contents and antioxidant activity of BSGs.

BSGs	TPC (mg/g d.m.)	Antioxidant Activity (mg Trolox/g d.m.)	Phenolics (mg/g d.m.)										
			Gallic Acid	Vanillic Acid	Caffeic Acid	*p*-Coumaric Acid	4-Hydroxybenzoic Acid	Ferulic Acid	Epicatechin	Epicatechin Gallate	Resveratrol	Quercetin	Kaempferol
Interactive effects (Type of BSG* Geographical origin of Cereal Mixtures)													
Ri-D	3.129 ± 0.107 ^a^	2.88 ± 0.14 ^c^	0.016 ^c^	0.010	0.178 ^bc^	0.018 ^b^	0.001 ^b^	0.047 ^d^	0.164 ^bc^	0.0004 ^e^	0.031 ^c^	0.029 b	1.018 ± 0.022 ^c^
Ri-S	3.114 ± 0.326 ^a^	2.89 ± 0.11 ^c^	0.017 ^d^	0.010	0.016 ^abc^	0.018 ^b^	n.d.^a^	0.046 ^a^	0.160 ^a^	0.002 ^b^	0.030 ^b^	0.029 ^a^	0.914 ± 0.069 ^b^
Da-D	3.664 ± 0.085 ^a^	1.24 ± 1.00 ^ab^	0.016 ^c^	0.010	0.014 ± 0.002 ^a^	0.017 ^a^	0.001 ^b^	0.046 ^bc^	0.165 ^c^	0.003 ^c^	0.307 ^a^	0.029 ^a^	1.382 ± 0.020 ^d^
Da-S	3.274 ± 0.187 ^a^	1.39 ± 0.20 ^ab^	0.015 ^a^	0.010	0.018 ^cd^	0.017 ^a^	0.003 ^d^	0.046 ^c^	0.163 ^b^	0.001 ^a^	0.031 ^b^	0.029 ^b^	0.900 ± 0.179 ^b^
Em-D	4.868 ± 0.821 ^b^	0.64 ± 0.10 ^a^	0.017 ^d^	0.010	0.020 ^d^	0.019 ^c^	0.005 ^e^	0.048 ^e^	0.168 ^d^	0.002 ^c^	0.032 ^e^	0.031 ^d^	1.807 ± 0.016 ^e^
Em-S	4.789 ± 0.191 ^b^	1.96 ± 0.11 ^bc^	0.015 ^b^	0.006 ± 0.004	0.015 ^ab^	0.018 ^c^	0.002 ^c^	0.048 ^e^	0.1645 ^c^	0.003 ^d^	0.032 ^d^	0.031 ^c^	0.795 ± 0.039 ^a^
Significance	*	*	*	n.s.	*	*	*	*	*	*	*	*	*
Single Effect (Type of BSG)													
Ri	3.122 ^a^	2.88 ^b^	0.017 ^c^	0.010	0017 a^b^	0.018 ^b^	0.001 ^a^	0.047 ^b^	0.162 ^a^	0.003 ^b^	0.031 ^b^	0.030 ^a^	0.966 ^a^
Da	3.469 ^b^	1.32 ^a^	0.015 ^a^	0.010	0.016 ^a^	0.017 ^a^	0.002 ^b^	0.046 ^a^	0.164 ^b^	0.002 ^a^	0.030 ^a^	0.030 ^a^	1.116 ^b^
Em	4.829 ^c^	1.30 ^a^	0.016 ^b^	0.010	0.018 ^b^	0.019 ^c^	0.003 ^c^	0.048 ^c^	0.166 ^c^	0.003 ^b^	0.032 ^c^	0.031 ^b^	1.301 ^c^
Significance	*	*	*	n.s.	*	*	*	*	*	*	*	*	*
Single Effect (Geographical Origin of Cereal Mixtures)													
D	3.8870	1.59	0.017 ^b^	0.010	0.017	0.018	0.002 ^b^	0.047 ^b^	0.165 ^b^	0.003 ^b^	0.032 ^b^	0.031 ^b^	1.386 ^b^
S	3.726	2.08	0.016 ^a^	0.010	0.017	0.018	0.001 ^a^	0.046 ^a^	0.162 ^a^	0.002 ^a^	0.031 ^a^	0.030 ^a^	0.870 ^a^
Significance	n.s.	n.s.	*	n.s.	n.s.	n.s.	*	*	*	*	*	*	*

In column, different letters indicate significant differences at *p* < 0.01; * *p* < 0.01; n.s.: not significant; n.d.: not detected.

**Table 5:** Effects of type of BSG and amounts of BSG-gluten in the formulation on physical and chemical characteristics of the functional breads.

Bread Samples	Crust			Crumb			Crust Moisture %	Crumb Moisture %	Ash (%)	UP%
	L*	a*	b*	L*	a*	b*				
Control	57.0 ± 2.5 ^def^	16.6 ± 1.2 ^a^	47.0 ± 1.1 ^g^	69.2 ± 5.6 ^g^	2.6 ± 0.5 ^a^	29.4 ± 1.6 ^ab^	10.5 ± 1.1 ^cde^	24.5 ± 2.4 ^c^	3.1 ^a^	41.00 ± 0.68 ^ef^
Interactive effects (Type of BSG* Geographical origin of Cereal Mixtures* Amount BSG-gluten)										
Ri-D-20 G3	43.8 ± 3.3 ^a^	19.3 ± 0.8 ^d^	37.9 ± 1.5 ^bc^	46.7 ± 2.8 ^a^	6.7 ± 0.4 ^b^	29.2 ± 1.2 ^a^	7.8 ± 0.7 ^ab^	16.9 ± 1.8 ^a^	3.3 ^b–e^	38.98 ± 0.84 ^cde^
Ri-D-25 G4	53.8 ± 2.3 ^bc^	17.3 ± 0.7 ^b^	39.6 ± 0.8 ^bc^	58.0 ± 5.1 ^ef^	10.4 ± 0.5 ^g^	35.1 ± 1.8 ^g^	11.2 ± 1.1 ^de^	23.6 ± 0.8 ^bc^	3.4 ^efg^	40.26 ± 0.52 ^def^
Ri-S-20 G3	54.5 ± 4.1 ^cd^	16.3 ± 1.4 ^ab^	40.0 ± 2.4 ^cd^	53.7 ± 2.7 ^bd^	8.1 ± 2.6 ^de^	33.7 ± 1.5 ^d–g^	10.1 ± 1.0 ^b–e^	21.8 ± 2.7 ^bc^	3.2 ^b^	42.07 ± 0.15 ^f^
Ri-S-25 G4	60.0 ± 3.9 ^f^	16.1 ± 1.9 ^a^	42.8 ± 2.9 ^ef^	53.0 ± 2.4 ^b^	9.0 ^def^	33.8 ± 0.9 ^d–g^	9.7 ± 0.9 ^a–e^	20.7 ± 1.3 ^abc^	3.3 ^bcd^	41.02 ± 0.45 ^ef^
Da-D-20 G3	55.1 ± 1.7 ^cd^	20.7 ± 0.5 ^e^	41.5 ± 0.5 ^de^	56.4 ± 5.1 ^b–f^	8.7 ± 1.6 ^de^	33.3 ± 2.7 ^def^	8.3 ± 1.8 ^abc^	18.9 ± 1.5 ^ab^	3.3 ^c–f^	39.19 ± 0.56 ^de^
Da-D-25 G4	56.7 ± 3.2 ^cde^	16.9 ± 1.1 ^a^	40.6 ± 0.8 ^cd^	58.3 ± 2.1 ^f^	10.0 ± 0.5 ^fg^	37.0 ± 1.1 ^g^	9.7 ± 1.7 ^a–e^	23.6 ± 0.2 ^bc^	3.4 ^def^	36.47 ± 1.29 ^ab^
Da-S-20 G3	62.4 ± 1.8 ^f^	16.8 ± 0.6 ^a^	45.9 ± 1.0 ^g^	53.1 ± 2.7 ^bc^	7.9 ± 0.3 ^ce^	32.0 ± 1.0 ^cd^	10.4 ± 1.5 ^b–e^	24.8 ± 1.5 ^c^	3.2 ^bc^	39.18 ± 0.06 ^de^
Da-S-25 G4	51.3 ± 2.0 ^b^	21.1 ± 1.4 ^e^	40.5 ± 1.1 ^cd^	58.4 ± 2.7 ^f^	9.0 ± 1.2 ^ef^	34.8 ± 1.9 ^fg^	9.2 ± 0.4 ^a–d^	23.2 ± 2.7 ^bc^	3.4 ± 0.1 ^def^	39.67 ± 1.09 ^de^
Em-D-20 G3	59.3 ± 2.4 ^ef^	16.9 ± 0.6 ^a^	44.0 ± 1.8 ^f^	57.1 ± 1.8 ^def^	6.8 ± 0.8 ^bc^	33.8 ± 1.9 ^efg^	12.1 ± 1.6 ^e^	20.9 ± 0.5 ^abc^	3.4 ^fg^	38.18 ± 0.01 ^bcd^
Em-D-25 G4	54.2 ± 1.6 ^bcd^	19.5 ± 07 ^d^	39.1 ± 1.5 ^b^	56.9 ± 2.9 ^b–f^	7.8 ± 0.4 ^bcd^	32.5 ± 1.0 ^cde^	10.7 ± 0.2 ^c–e^	24.6 ± 4.0 ^c^	3.5 ^h^	36.82 ± 1.20 ^abc^
Em-S-20 G3	59.1 ± 1.9 ^ef^	18.4 ± 0.5 ^c^	43.1 ± 1.8 ^ef^	57.0 ± 2.2 ^c–f^	7.9 ± 0.3 ^ce^	33.8 ± 0.4 ^efg^	9.2 ± 0.7 ^a–d^	17.0 ± 0.3 ^a^	3.5 ± 0.1 ^gh^	35.91 ± 0.38 ^a^
Em-S-25 G4	44.5 ± 1.9 ^a^	19.3 ± 0.5 ^d^	34.5 ± 1.4 ^a^	54.1 ± 2.3 ^b–e^	7.9 ± 0.3 ^ce^	31.0 ± 0.9 ^bc^	7.3 ± 1.3 ^a^	18.7 ± 2.0 ^ab^	3.6 ^i^	38.26 ± 0.55 ^bcd^
Significance	*	*	*	*	*	*	*	*	*	*
Single Effect (Type of BSG)										
Ri	53.0 ^a^	17.2 ^a^	40.1 ^a^	52.8 ^a^	8.6 ^b^	32.6 ^a^	9.7	20.8 ^ab^	3.3 ^a^	40.58 ^c^
Da	56.4 ^b^	18.5 ^b^	42.1 ^b^	56.6 ^b^	8.9 ^b^	34.3 ^b^	9.4	22.6 ^b^	3.3 ^a^	38.63 ^b^
Em	54.3 ^a^	18.8 ^b^	40.2 ^a^	56.3 ^b^	7.6 ^a^	32.8 ^a^	9.8	20.3 ^a^	3.5 ^b^	37.29 ^a^
Significance	*	*	*	*	*	*	n.s.	*	*	*
Single Effect (Geographical Origin of Cereal Mixtures)										
D	53.8	18.4	40.4	55.6	8.4	33.5	10.3	21.4	3.4	38.32
S	55.3	18.0	41.1	54.8	8.3	33.2	9.3	21.0	3.4	39.35
Significance	n.s.	n.s.	n.s.	n.s.	n.s.	n.s.	n.s.	n.s.	n.s.	n.s.
Single Effect (Amount BSG-gluten)										
20 G3	55.7 ^b^	18.1	41.1 ^b^	54.0 ^a^	7.7 ^a^	32.6 ^a^	9.6	20.1 ^a^	3.3 ^a^	38.92
25 G4	53.4 ^a^	18.2	39.5 ^a^	56.4 ^b^	9.0 ^b^	34.0 ^b^	9.6	22.4 ^b^	3.4 ^b^	38.75
Significance	*	n.s.	*	*	*	*	n.s.	*	*	n.s.

In column, different letters indicate significant differences at *p* < 0.01; * *p* < 0.01; n.s.: not significant.

**Table 6:** Effects of type of BSG, geographical origin, and amounts of BSG-gluten in the formulation on antioxidant and fibre contents of the functional breads.

Bread Samples	TPC (mg/g d.m.)	Antioxidant Activity (mg Trolox/g d.m.)	Gallic Acid	Vanillic Acid	Caffeic Acid	*p*-Coumaric Acid	Sinapic Acid	Epigallocatechin Gallate	IDF%	SDF%
Control	1.555 ± 0.158 ^a^	0.36 ± 0.02 ^a^	0.028 ^a^	n.d. ^a^	n.d. ^a^	0.027 ^b^	n.d. ^a^	n.d. ^a^	2.05 ^a^	0.63 ^k^
Interactive effects (Type of BSG* Geographical origin of Cereal Mixtures* Amount BSG-gluten)										
Ri-D-20 G3	1.793 ± 0.183 ^b^	0.67 ± 0.16 ^b^	0.040 ^de^	n.d. ^a^	0.018 ^f^	n.d.	n.d.^a^	n.d. ^a^	4.17 ^b^	0.33 ^f^
Ri-D-25 G4	2.066 ± 0.159 ^bcd^	0.97 ± 0.12 ^bc^	0.029 ^ab^	n.d. ^a^	0.018 ^f^	n.d.	n.d.^a^	n.d. ^a^	5.98 ^g^	0.05 ^b^
Ri-S-20 G3	1.881 ± 0.117 ^bc^	1.48 ± 0.18 ^cd^	0.029 ^ab^	n.d. ^a^	0.018 ^f^	n.d.	n.d.^a^	n.d. ^a^	5.08 ^d^	0.20 ^d^
Ri-S-25 G4	2.300 ± 0.135 ^b-e^	2.12 ± 0.46 ^ef^	0.049 ^f^	n.d. ^a^	0.018 ^f^	n.d.	n.d.^a^	n.d. ^a^	6.76 ^k^	0.004 ^a^
Da-D-20 G3	1.888 ± 0.086 ^bc^	2.00 ± 0.10 ^de^	0.031 ± 0.003 ^ab^	0.015 ^b^	0.017 ^d^	n.d.	0.017 ^b^	n.d. ^a^	5.25 ^e^	0.30 ^e^
Da-D-25 G4	2.488 ± 0.583 ^b-e^	2.53 ± 0.14 ^e-g^	0.035 ^bc^	0.017 ^b-d^	0.017 ^d^	n.d.	0.033 ^c^	n.d. ^a^	6.15 ^h^	0.43 ^h^
Da-S-20 G3	1.951 ± 0.106 ^b-d^	1.51 ± 0.09 ^cd^	0.028 ^a^	0.016 ^bc^	0.015 ^b^	n.d.	0.018 ^b^	n.d. ^a^	4.49 ^c^	0.52 ^i^
Da-S-25 G4	2.833 ± 0.772 ^e^	2.93 ± 0.39 ^gh^	0.040 ^de^	0.018 ^de^	0.017 ^d^	n.d.	0.035 ± 0.002 ^cd^	0.010 ± 0.001 ^b^	5.36 ^f^	1.56 ^n^
Em-D-20 G3	2.169 ± 0.246 ^b-e^	2.61 ± 0.37 ^fg^	0.041 ^e^	0.018 ^de^	0.016 ^c^	n.d.	0.037 ± 0.001 ^d^	0.022 ± 0.002 ^c^	6.30 ^i^	0.78 ^m^
Em-D-25 G4	2.584 ± 0.289 ^de^	3.45 ± 0.47 ^h^	0.048 ± 0.005 ^f^	0.020 ^e^	0.018 ^f^	n.d.	0.042 ^e^	0.030 ± 0.004 ^d^	6.84 ^l^	0.08 ^c^
Em-S-20 G3	2.550 ± 0.089 ^c-e^	2.87 ± 0.43 ^g^	0.046 ^ef^	0.025 ^g^	0.017 ^d^	n.d.	0.019 ± 0.001 ^b^	n.d.^a^	6.76 ^k^	0.40 ^g^
Em-S-25 G4	2.453 ± 0.139 ^b-e^	2.63 ± 0.06 ^fg^	0.044 ± 0.004 ^ef^	0.018 ± 0.001 ^ef^	0.017 ^d^	n.d.	0.019 ^b^	0.002 ^a^	7.15 ^m^	0.70 ^l^
Significance	*	*	*	*	*		*	*	*	*
Single Effect (Type of BSG)										
Ri	2.009 ^a^	1.310 ^a^	0.037 ^b^	n.d. ^a^	0.018 ^c^	n.d.	n.d. ^a^	n.d. ^a^	5.50 ^b^	0.15 ^a^
Da	2.290 ^ab^	2.243 ^b^	0.033 ^a^	0.017 ^b^	0.016 ^a^	n.d.	0.026 ^b^	0.003 ^b^	5.31 ^a^	0.70 ^c^
Em	2.439 ^b^	2.891 ^c^	0.045 ^c^	0.020 ^c^	0.017 ^b^	n.d.	0.029 ^c^	0.013 ^c^	6.76 ^c^	0.49 ^b^
Significance	*	*	*	*	*		*	*	*	*
Single Effect (Geographical Origin of Cereal Mixtures)										
D	2.165	2.039	0.037	0.012	0.017	n.d.	0.021 ^b^	0.009 ^b^	5.78 ^a^	0.33 ^a^
S	2.327	2.257	0.039	0.013	0.017	n.d.	0.015 ^a^	0.002 ^a^	5.93 ^b^	0.56 ^b^
Significance	n.s.	n.s.	n.s.	n.s.	n.s.		*	*	*	*
Single Effect (Amount BSG-gluten)										
20 G3	2.039 ^a^	1.859 ^a^	0.036 ^a^	0.012	0.016 ^a^	n.d.	0.015 ^a^	0.004 ^a^	5.34 ^a^	0.42 ^a^
25 G4	2.453 ^b^	2.437 ^b^	0.041 ^b^	0.012	0.017 ^b^	n.d.	0.021 ^b^	0.007 ^b^	6.37 ^b^	0.47 ^b^
Significance	*	*	*	n.s.	*		*	*	*	*

In column, different letters indicate significant differences at *p* < 0.01; * *p* < 0.01; n.s.: not significant; n.d.: not detected.

**Table 7:** Effects of type of BSG, geographical origin, and amounts of BSG-gluten in the formulation on structural characteristics of the functional breads.

Bread Samples	Minimum Slice Height (cm)	Maximum Slice Height (cm)	Crumb Specific Volume (cm^3^/g)	N. pores/cm^2^	Average Pore Size (mm^2^)	Porosity %	Pore Circularity
Control	9.36 ± 0.24 ^d^	10.14 ± 0.17 ^d^	2.64 ^b^	0.73 ± 0.02 ^cd^	0.10 ^a^	34.9 ± 1.3 ^a^	0.800 ± 0.007 ^a-d^
Interactive effects (Type of BSG* Geographical origin of Cereal Mixtures* Amount BSG-gluten)							
Ri-D-20 G3	8.01 ± 0.6 ^ab^	8.97 ± 0.22 ^abc^	2.37 ^ab^	0.59 ± 0.07 ^bc^	0.18 ± 0.04 ^abc^	39.5 ^a-d^	0.813 ± 0.007 ^b-e^
Ri-D-25 G4	7.94 ± 0.24 ^ab^	8.39 ± 0.20 ^a^	2.29 ^ab^	0.70 ± 0.07 ^cd^	0.14 ± 0.05 ^ab^	35.8 ± 1.8 ^a^	0.835 ± 0.013 ^e^
Ri-S-20 G3	8.52 ± 0.65 ^a-d^	9.11 ± 0.59 ^abc^	2.47 ^ab^	0.34 ± 0.03 ^a^	0.30 ^d^	36.0 ± 1.9 ^ab^	0.774 ± 0.029 ^a^
Ri-S-25 G4	8.38 ± 0.57 ^abc^	9.11 ± 0.55 ^abc^	2.50 ^ab^	0.39 ± 0.07 ^a^	0.24 ± 0.05 ^bcd^	36.6 ± 1.8 ^ab^	0.788 ± 0.012 ^ab^
Da-D-20 G3	8.43 ± 0.50 ^a-d^	9.26 ± 0.29 ^bc^	2.11 ± 0.9 ^a^	0.69 ± 0.10 ^cd^	0.14 ± 0.05 ^ab^	35.2 ± 2.5 ^a^	0.816 ± 0.005 ^b-e^
Da-D-25 G4	8.14 ± 0.26 ^abc^	9.21 ± 0.20 ^abc^	2.45 ^ab^	0.66 ± 0.09 ^cd^	0.16 ± 0.05 ^ab^	38.4 ± 2.0 ^a-d^	0.825 ± 0.010 ^de^
Da-S-20 G3	7.72 ± 0.84 ^a^	8.97 ± 0.71 ^abc^	2.32 ^ab^	0.69 ± 0.07 ^cd^	0.16 ± 0.05 ^ab^	38.2 ± 2.2 ^abc^	0.835 ± 0.026 ^e^
Da-S-25 G4	7.99 ± 0.45 ^ab^	8.48 ± 0.22 ^ab^	2.35 ^ab^	0.75 ± 0.04 ^cd^	0.10 ^a^	36.4 ± 3.8 ^ab^	0.817 ± 0.013 ^cde^
Em-D-20 G3	8.58 ± 0.43 ^a-d^	8.89 ± 0.82 ^abc^	2.42 ^ab^	0.82 ± 0.17 ^d^	0.16 ± 0.05 ^ab^	43.1 ± 2.9 ^de^	0.819 ± 0.006 ^cde^
Em-D-25 G4	8.84 ± 1.21 ^bcd^	9.67 ± 0.92 ^cd^	2.64 ^b^	0.33 ± 0.08 ^a^	0.32 ± 0.08 ^d^	42.6 ± 0.1 ^cde^	0.807 ± 0.015 ^b-e^
Em-S-20 G3	8.46 ± 0.31 ^a-d^	9.72 ± 0.41 ^cd^	2.52 ^ab^	0.47 ± 0.22 ^ab^	0.24 ± 0.11 ^bcd^	40.8 ± 3.6 ^bcd^	0.791 ± 0.017 ^abc^
Em-S-25 G4	9.08 ± 0.18 ^cd^	9.58 ± 0.21 ^cd^	2.27 ^ab^	0.40 ± 0.11 ^a^	0.28 ± 0.08 ^cd^	46.7 ± 6.2 ^e^	0.829 ± 0.029 ^e^
Significance	*	*	*	*	*	*	*
Single Effect (Type of BSG)							
Ri	8.21 ^a^	8.89 ^a^	2.41	0.50 ^a^	0.21 ^b^	36.9 ^a^	0.80
Da	8.01 ^a^	8.98 ^a^	2.30	0.70 ^b^	0.14 ^a^	37.0 ^a^	0.82
Em	8.74 ^b^	9.46 ^b^	2.46	0.50 ^a^	0.25 ^b^	43.3 ^b^	0.81
Significance	*	*	n.s.	*	*	*	n.s.
Single Effect (Geographical Origin of Cereal Mixtures)							
D	8.31	9.06	2.38	0.63 ^b^	0.18	39.1	0.82
S	8.36	9.16	2.40	0.51 ^a^	0.22	39.1	0.81
Significance	n.s.	n.s.	n.s.	*	n.s.	n.s.	n.s.
Single Effect (Amount BSG-gluten)							
20 G3	8.29	9.15	2.37	0.60 ^b^	0.20	38.8	0.81
25 G4	8.39	9.07	2.42	0.54 ^a^	0.20	39.4	0.82
Significance	n.s.	n.s.	n.s.	*	n.s.	n.s.	n.s.

In column, different letters indicate significant differences at *p* < 0.01; * *p* < 0.01; n.s.: not significant.

**Table 8:** Effects of type of BSG, geographical origin, and amounts of BSG-gluten in the formulation on sensorial parameters of the functional breads.

Bread Samples	Visual Characteristic					Smell						Taste			Tactile Characteristics/Texture					Overall Quality
	Crust		Crumb			Crust and Crumb				Crust	Crumb	Crumb			Crust		Crumb			
	Colour	Thickness	Colour	Pore Size	Development	Overall Intensity	Freshly Baked Bread	Wheat	Malty	Toasty	Yeast	Sweetness	Saltiness	Acidity	Hardness	Crispiness	Resistance to Chewing	Cohesiveness	Fibrousness	
Control	0.5 ± 0.1 ^a^	4.5 ± 0.6 ^ab^	0.5 ± 0.1 ^a^	2.5 ± 0.6 ^ab^	8.5 ± 0.6 ^c^	4.5 ± 0.6 ^a^	4.5 ± 0.6 ^de^	0.5 ± 0.1 ^a^	1.5 ± 0.3 ^bc^	0.5 ± 0.1 ^a^	0.5 ± 0.1 ^a^	6.5 ± 0.6 ^ab^	7.5 ± 0.6 ^f^	2.5 ± 0.2 ^c^	3.5 ± 0.6 ^a^	4.5 ± 0.6 ^bc^	0 ^a^	7.5 ± 0.6 ^b^	0 ^a^	7.5 ± 0.6 ^c^
Interactive effects (Type of BSG* Geographical origin of Cereal Mixtures* Amount BSG-gluten)																				
Ri-D-20 G3	6.5 ± 0.6 ^b^	4.5 ± 0.6 ^ab^	6.5 ± 0.6 ^cd^	3.5 ± 0.6 ^b^	6.5 ± 0.6 ^ab^	7.5 ± 0.6 ^c^	2.5 ± 0.6 ^bc^	2.5 ± 0.6 ^bc^	3.5 ± 0.6 ^d^	2.5 ± 0.1 ^b^	0.5 ± 0.1 ^a^	6.5 ± 0.6 ^ab^	4.5 ± 0.6 ^de^	1.5 ± 0.3 ^bc^	4.5 ± 0.6 ^ab^	1.5 ± 0.2 ^a^	1.5 ± 0.1 ^bc^	6.5 ± 0.6 ^b^	3.5 ± 0.6 ^de^	7.5 ± 0.6 ^c^
Ri-D2-5 G4	6.5 ± 0.6 ^b^	3.5 ± 0.6 ^a^	7.5 ± 0.6 ^d^	3.5 ± 0.6 ^b^	6.5 ± 0.6 ^ab^	6.5 ± 0.6 ^bc^	2.5 ± 0.6 ^bc^	3.5 ± 0.6 ^cd^	3.5 ± 0.6 ^d^	1.5 ± 0.1 ^ab^	2.5 ± 0.3 ^bc^	5.5 ± 0.6 ^a^	4.5 ± 0.6 ^de^	0.5 ± 0.2 ^ab^	3.5 ± 0.6 ^a^	1.5 ± 0.1 ^a^	1.5 ± 0.2 ^bc^	6.5 ± 0.6 ^b^	2.5 ± 0.2 ^cd^	7.5 ± 0.6 ^c^
RiS20 G3	7.5 ± 0.6 ^bc^	4.5 ± 0.^6 ab^	7.5 ± 0.6 ^d^	4.5 ± 0.6 ^c^	6.5 ± 0.6 ^ab^	7.5 ± 0.6 ^c^	2.5 ± 0.6 ^bc^	1.5 ± 0.6 ^ab^	1.5 ± 0.6 ^bc^	1.5 ± 0.1 ^ab^	1.5 ± 0.1 ^ab^	6.5 ± 0.6 ^ab^	3.5 ± 0.5 ^cd^	0.5 ± 0.1 ^ab^	3.5 ± 0.6 ^a^	4.5 ± 0.6 ^bc^	1.5 ± 0.4 ^bc^	7.5 ± 0.6 ^b^	2.5 ± 0.2 ^cd^	7.5 ± 0.6 ^c^
Ri-S-25 G4	6.5 ± 0.6 ^b^	3.5 ± 0.6 ^a^	7.5 ± 0.6 ^d^	3.5 ± 0.6 ^b^	7.5 ± 0.6 ^bc^	6.5 ± 0.6 ^bc^	5.5 ± 0.6 ^e^	4.5 ± 0.6 ^de^	3.5 ± 0.6 ^d^	1.5 ± 0.2 ^ab^	5.5 ± 0.6 ^d^	5.5 ± 0.6 ^a^	3.5 ± 0.6 ^cd^	1.5 ± 0.2 ^bc^	3.5 ± 0.5 ^a^	1.5 ± 0.2 ^a^	2.5 ± 0.2 ^cd^	7.5 ± 0.6 ^b^	0.5 ± 0.1 ^ab^	7.5 ± 0.6 ^c^
Da-D-20 G3	6.5 ± 0.6 ^b^	5.5 ± 0.6 ^b^	6.5 ± 0.6 ^cd^	4.5 ± 0.6 ^c^	5.5 ± 0.6 ^a^	6.5 ± 0.6 ^bc^	5.5 ± 0.6 ^e^	0.5 ± 0.1 ^a^	0.5 ± 0.1 ^ab^	1.5 ± 0.2 ^ab^	1.5 ± 0.1 ^ab^	5.5 ± 0.6 ^a^	2.5 ± 0.3 ^bc^	1.5 ± 0.3 ^bc^	5.5 ± 0.6 ^bc^	0.5 ± 0.2 ^a^	3.5 ± 0.6 ^d^	4.5 ± 0.1 ^a^	0.5 ± 0.1 ^ab^	5.5 ± 0.6 ^ab^
Da-D-25 G4	6.5 ± 0.6 ^b^	4.5 ± 0.6 ^ab^	6.5 ± 0.6 ^cd^	2.5 ± 0.6 ^ab^	6.5 ± 0.6 ^ab^	6.5 ± 0.6 ^bc^	1.5 ± 0.3 ^ab^	2.5 ± 0.6 ^bc^	0.5 ± 0.1 ^ab^	1.5 ± 0.5 ^ab^	2.5 ± 0.6 ^bc^	5.5 ± 0.6 ^a^	3.5 ± 0.6 ^cd^	0.5 ± 0.2 ^ab^	5.5 ± 0.6 ^bc^	3.5 ± 0.6 ^b^	1.5 ± 0.6 ^bc^	6.5 ± 0.6 ^b^	0.5 ± 0.1 ^ab^	7.5 ± 0.6 ^c^
Da-S-20 G3	6.5 ± 0.6 ^b^	5.5 ± 0.6 ^b^	6.5 ± 0.6 ^cd^	3.5 ± 0.6 ^b^	5.5 ± 0.6 ^a^	6.5 ± 0.6 ^bc^	3.5 ± 0.6 ^cd^	0.5 ± 0.1 ^a^	1.5 ± 0.4 ^bc^	2.5 ± 0.5 ^b^	0.5 ± 0.1 ^a^	5.5 ± 0.6 ^a^	4.5 ± 0.5 ^de^	0.5 ± 0.1 ^ab^	4.5 ± 0.6 ^ab^	5.5 ± 0.6 ^cd^	2.5 ± 0.3 ^cd^	6.5 ± 0.6 ^b^	0.5 ± 0.1 ^ab^	7.5 ± 0.6 ^c^
Da-S-25 G4	7.5 ± 0.6 ^bc^	5.5 ± 0.6 ^b^	6.5 ± 0.6 ^cd^	2.5 ± 0.6 ^ab^	5.5 ± 0.6 ^a^	7.5 ± 0.6 ^c^	2.5 ± 0.4 ^bc^	2.5 ± 0.3 ^bc^	2.5 ± 0.2 ^cd^	1.5 ± 0.3 ^ab^	2.5 ± 0.5 ^bc^	7.5 ± 0.6 ^b^	5.5 ± 0.6 ^e^	1.5 ± 0.2 ^bc^	5.5 ± 0.5 ^bc^	1.5 ± 0.6 ^a^	2.5 ± 0.6 ^cd^	6.5 ± 0.6 ^b^	1.5 ± 0.6 ^bc^	6.5 ± 0.5 ^bc^
Em-D-20 G3	7.5 ± 0.6 ^bc^	3.5 ± 0.6 ^a^	4.5 ± 0.6 ^b^	2.5 ± 0.6 ^ab^	7.5 ± 0.6 ^bc^	4.5 ± 0.6 ^a^	1.5 ± 0.1 ^ab^	3.5 ± 0.6 ^cd^	0.5 ± 0.1 ^ab^	0.5 ± 0.1 ^a^	1.5 ± 0.3 ^ab^	6.5 ± 0.6 ^ab^	3.5 ± 0.6 ^cd^	0.5 ± 0.1 ^ab^	3.5 ± 0.5 ^a^	6.5 ± 0.6 ^d^	0.5 ± 0.1 ^ab^	6.5 ± 0.1 ^b^	5.5 ± 0.6 ^f^	5.5 ± 0.5 ^ab^
Em-D-25 G4	8.5 ± 0.6 ^c^	3.5 ± 0.6 ^a^	7.5 ± 0.6 ^d^	2.5 ± 0.6 ^ab^	7.5 ± 0.6 ^bc^	6.5 ± 0.6 ^bc^	5.5 ± 0.6 ^e^	5.5 ± 0.6 ^e^	0.5 ± 0.6 ^ab^	1.5 ± 0.2 ^ab^	1.5 ± 0.2 ^ab^	5.5 ± 0.4 ^a^	1.5 ± 0.3 ^ab^	1.5 ± 0.3 ^bc^	6.5 ± 0.5 ^c^	4.5 ± 0.4 ^bc^	5.5 ± 0.6 ^e^	6.5 ± 0.6 ^b^	3.5 ± 0.6 ^de^	4.5 ± 0.4 ^a^
Em-S-20 G3	6.5 ± 0.6 ^b^	4.5 ± 0.6 ^ab^	5.5 ± 0.6 ^bc^	1.5 ± 0.4 ^a^	6.5 ± 0.6 ^ab^	5.5 ± 0.6 ^ab^	3.5 ± 0.6 ^cd^	5.5 ± 0.6 ^e^	0.5 ± 0.2 ^ab^	0.5 ± 0.1 ^a^	3.5 ± 0.2 ^c^	6.5 ± 0.4 ^ab^	1.5 ± 0.3 ^ab^	1.5 ± 0.3 ^bc^	4.5 ± 0.5 ^ab^	6.5 ± 0.6 ^d^	1.5 ± 0.2 ^bc^	7.5 ± 0.6 ^b^	3.5 ± 0.3 ^de^	6.5 ± 0.5 ^bc^
Em-S-25 G4	7.5 ± 0.6 ^bc^	4.5 ± 0.6 ^ab^	5.5 ± 0.6 ^bc^	1.5 ± 0.3 ^a^	6.5 ± 0.6 ^ab^	4.5 ± 0.6 ^a^	0.5 ± 0.1 ^a^	4.5 ± 0.6 ^de^	0 ^a^	0.5 ± 0.1 ^a^	1.5 ± 0.3 ^ab^	5.5 ± 0.5 ^a^	0.5 ± 0.1 ^a^	0 ^a^	3.5 ± 0.4 ^a^	5.5 ± 0.5 ^cd^	1.5 ± 0.3 ^bc^	6.5 ± 0.6 ^b^	4.5 ± 0.5 ^ef^	4.5 ± 0.6 ^a^
Significance	*	*	*	*	*	*	*	*	*	*	*	*	*	*	*	*	*	*	*	*
Single Effect (Type of BSG)																				
Ri	6.^7 a^	4.0 ^a^	7.2 ^c^	3.7 ^c^	6.7 ^b^	7.0 ^b^	3.2	3.0 ^b^	3.0 ^c^	1.7 ^b^	2.5 ^b^	6.0	4.0 ^b^	1.0	3.7 ^a^	2.5 ^a^	1.7 ^a^	7.0 ^b^	2.2 ^b^	7.5 ^c^
Da	6.7 ^a^	5.2 ^b^	6.5 ^b^	3.2 ^b^	5.7 ^a^	6.7 ^b^	3.2	1.5 ^a^	1.2 ^b^	1.7 ^b^	1.7 ^a^	6.0	4.0 ^b^	1.0	5.2 ^c^	2.7 ^a^	2.5 ^b^	6.0 ^a^	0.7 ^a^	6.7 ^b^
Em	7.5 ^b^	4.0 ^a^	5.7 ^a^	2.0 ^a^	7.0 ^b^	5.2 ^a^	2.7	4.7 ^c^	0.4 ^a^	0.7 ^a^	2.0 ^ab^	6.0	1.7 ^a^	0.9	4.5 ^b^	5.7 ^b^	2.2 ^bc^	6.7 ^b^	4.2 ^c^	5.2 ^a^
Significance	*	*	*	*	*	*	n.s.	*	*	*	*	n.s.	*	n.s.	*	*	*	*	*	*
Single Effect (Geographical Origin of Cereal Mixtures)																				
D	7	4.2	6.5	3.2	6.7	6.3	3.1	3.0	1.5	1.5	1.7 ^a^	5.8	3.3	1.0	4.8 ^b^	3.0 ^a^	2.3	6.2 ^a^	2.7	6.3 ^a^
S	7	4.7	6.5	2.8	6.3	6.3	3.0	3.2	1.6	1.3	2.5 ^b^	6.2	3.2	0.9	4.2 ^a^	4.2 ^b^	2.0	7 ^b^	2.2	6.7 ^b^
Significance	n.s.	n.s.	n.s.	n.s.	n.s.	n.s.	n.s.	n.s.	n.s.	n.s.	*	n.s.	n.s.	n.s.	*	*	n.s.	*	n.s.	*
Single effect (Amount BSG-gluten)																				
20 G3	6.8	4.7	6.2	3.3	6.3	6.3	3.2	2.3 ^a^	1.3	1.5	1.5 ^a^	6.2	3.3	1.0	4.3 ^a^	4.2 ^b^	1.8 ^a^	6.5	2.7	6.7 ^b^
25 G4	7.2	4.2	6.8	2.7	6.7	6.3	3.0	3.8 ^b^	1.7	1.3	2.7 ^b^	5.8	3.2	0.9	4.7 ^b^	3.0 ^a^	2.5 ^b^	6.7	2.2	6.3 ^a^
Significance	n.s.	n.s.	n.s.	n.s.	n.s.	n.s.	n.s.	*	n.s.	n.s.	*	n.s.	n.s.	n.s.	*	*	*	n.s.	n.s.	*

In column, different letters indicate significant differences at *p* < 0.01; * *p* < 0.01; n.s.: not significant.

## Data Availability

No new data were created or analyzed in this study. Data sharing is not applicable to this article.

## Abbreviations

BSG	Brewers’s spent grain;
Ri	BSG deriving from mashing of a mixture of 65% barley malt and 35% unmalted soft wheat cv. Risciola;
Da	BSG deriving from mashing of a mixture of 65% barley malt and 35% unmalted durum wheat cv. Dauno III;
Em	BSG deriving from mashing of a mixture of 65% barley malt and 35% unmalted emmer;
D	Geographical origin from Daunia;
S	Geopraphical origin from Salento;
20G3 and 25G4	The first number is referred to the percentages of substitution of the Manitoba flour with BSG flour while the second one is referred to the percentages of gluten added calculated on the total amount of flours;
EVOO	Extra-virgin olive oil;
TPC	Total Phenolic Content;
tUPP%	Percentage of total unextractable polymeric proteins;
lUPP%	Percentage of large unextractable polymeric protein;
UP%	Percentage of unextractable (both monomeric and polymeric forms) proteins;
TDF	Total Dietary Fibres;
SDF	Soluble Dietary Fibres;
IDF	Insoluble Dietary Fibres.
